# Lin28 regulates thymic growth and involution and correlates with MHCII expression in thymic epithelial cells

**DOI:** 10.3389/fimmu.2023.1261081

**Published:** 2023-10-06

**Authors:** Shiyun Xiao, Wen Zhang, Jie Li, Nancy R. Manley

**Affiliations:** Department of Genetics, University of Georgia, Athens, GA, United States

**Keywords:** thymic epithelial cells, proliferation, thymic involution, MHCII, *Lin28a*, *Lin28b*, *Let-7g*, *Foxn1*

## Abstract

Thymic epithelial cells (TECs) are essential for T cell development in the thymus, yet the mechanisms governing their differentiation are not well understood. Lin28, known for its roles in embryonic development, stem cell pluripotency, and regulating cell proliferation and differentiation, is expressed in endodermal epithelial cells during embryogenesis and persists in adult epithelia, implying postnatal functions. However, the detailed expression and function of Lin28 in TECs remain unknown. In this study, we examined the expression patterns of *Lin28* and its target *Let-7g* in fetal and postnatal TECs and discovered opposing expression patterns during postnatal thymic growth, which correlated with FOXN1 and MHCII expression. Specifically, *Lin28b* showed high expression in MHCII^hi^ TECs, whereas *Let-7g* was expressed in MHCII^lo^ TECs. Deletion of *Lin28a* and *Lin28b* specifically in TECs resulted in reduced MHCII expression and overall TEC numbers. Conversely, overexpression of *Lin28a* increased total TEC and thymocyte numbers by promoting the proliferation of MHCII^lo^ TECs. Additionally, our data strongly suggest that *Lin28* and *Let-7g* expression is reliant on FOXN1 to some extent. These findings suggest a critical role for Lin28 in regulating the development and differentiation of TECs by modulating MHCII expression and TEC proliferation throughout thymic ontogeny and involution. Our study provides insights into the mechanisms underlying TEC differentiation and highlights the significance of Lin28 in orchestrating these processes.

## Introduction

*Lin28* is a small mRNA-binding protein that blocks processing of the *Let-7* miRNA. Both *Lin28* and *Let-7* were originally identified by their important roles in developmental timing control in *C. elegans* (1–3) and are evolutionarily conserved in sequence and function from worms to humans, which highlights the important role of these two ancient small molecules (4, 5). *Lin28* and *Let-7* are frequently expressed in opposing patterns during embryonic development and differentiation, in the early postnatal period, and in adults. In general, *Lin28* is expressed in stem and immature cells and is generally considered to promote pluripotency, while *Let-7* is expressed in mature cells and promotes differentiation. *Lin28* specifically suppresses the expression and function of *Let-7*, and in turn, *Let-7* feeds back on *Lin28* (6, 7). Importantly, both *Lin28* and *Let-7* also have the broad capability of regulating the expression of multiple genes. Thus, the *Lin28/Let-7* system forms a regulatory axis that plays critical roles in a wide range of physiological development and pathogenesis in many organisms, including humans (8, 9). These processes include stem cell self-renewal and differentiation (10, 11), glucose metabolism (8), tissue repair (12), hematopoietic progenitor reprogramming (13–15), and the attraction of inflammatory endothelium into cancer cells (16). Additionally, *Lin28* may act as an oncogene, while *Let-7* acts as a cancer suppressor in a variety of human cancers; an imbalance in the expression of both genes has been linked to the formation, progression, diagnosis, and therapy of multiple cancer types (16–19).

*Lin28/Let-7* has different expression patterns with regard to level, timing, and function during the fetal to postnatal period (20–23), with studies focused mostly on tissues of germline, ectodermal, and mesodermal origin (24). Less progress has been made on endodermal cell types; however, the general pattern of *Lin28* expression in progenitors and *Let-7* expression in more differentiated cells is also seen in the epithelia of the small intestine and the seminiferous epithelium of the testis (25, 26). *Lin28* has two orthologs in mammals, *Lin28a* and *Lin28b* (22, 23), which have divergent temporal and cellular expression patterns in the seminiferous epithelium, with LIN28a expressed in undifferentiated A cells and LIN28b in spermatid cells at a later stage (25, 26). *Let-7s* also show opposing profiles to that of *Lin28b* in this adult tissue (26). There are also temporal dynamics for *Lin28* expression; *Lin28a* mRNA levels peak in neonates and *Lin28b* in young adults during postnatal maturation (25, 26). *Lin28b* expression in the seminiferous epithelium in mice is also consistent with genome-wide association studies in which the *Lin28b* locus was associated with the timing of puberty at menarche and height in humans (27).

Although *Lin28b* (but not Lin28a) and *Let-7g* have been reported to be expressed in fetal and early postnatal thymocytes (13) and thymic pro B cells (28), their expression and function in thymic epithelial cells (TECs) is poorly characterized. *Let-7* has 12 homologs in mice (5, 29), of which only *Let-7g* and *Let-7f2* have relatively high expression levels in TECs (30). *Let-7g*, but not *Let-7f2*, also has predicted sites in the 3’ and 5’UTRs (untranslated regions) of the *Lin28a* and *Lin28b* genes (31, 32), suggesting that these genes form a negative regulatory loop. The thymic primordium initiates from the endoderm of the third pharyngeal pouches in mid-gestation (33). Endoderm-derived TECs are critical for most aspects of T cell development from thymocytes. FOXN1 is a key transcription factor required for TEC proliferation, differentiation, and homeostasis that is expressed in TECs from embryonic day 11.25 (E11.25) (33–36). The thymus also undergoes age-associated degeneration, called thymic involution, which is associated with reduced thymocyte number and T cell production. Current evidence suggests that this decline is initiated primarily due to the decline in *Foxn1* expression in TECs (37). However, the mechanisms by which FOXN1 regulates these various aspects of TEC biology have not been fully investigated.

In this study, we investigated the expression and function of *Lin28* and *Let-7* in TECs. *Lin28a* was expressed at very low levels or undetected in fetal and postnatal TECs. *Lin28b* and *Let-7g* were detected in TEC progenitors at E13.5; their expression increased from fetal to adult stages but then declined by 6 months of age, associated with the process of age-related thymic involution. However, their expression showed opposite patterns in relation to major histocompatibility complex (MHC) class II (MHCII)^hi^ and MHCII^lo^ TECs. *Lin28b* genes were highly expressed in MHCII^hi^ TECs and expressed at very low or undetectable levels in MHCII^lo^ TECs, while the *Let-7g* gene was highly expressed in MHCII^lo^ and expressed at very low levels in MHCII^hi^ TECs. Immunofluorescence staining showed that both *Lin28a* and *Lin28b* were differentially correlated with FOXN1 and MHCII expression. TEC-specific *Lin28a* gain and *Lin28a* and *Lin28b* loss of function showed that *Lin28a* promoted the proliferation of MHCII^lo^ TECs and that both *Lin28a* and *Lin28b* were required for maintaining MHCII expression in TECs. We also showed that *Foxn1* expression was correlated with both *Lin28b* and *Let-7g* expression during ontogeny and that reducing *Foxn1* expression in *Foxn1^Z/Z^* mice reduced *Lin28* and *Let-7g* expression. *Lin28b*, but not *Let-7g*, expression increased during thymic rebound induced by sex steroid ablation. In summary, our findings indicate that Lin28 and Let-7 may both modulate MHCII expression and regulate thymic growth and involution by controlling TEC proliferation in the postnatal thymus.

## Materials and methods

### Mice

C57BL6/J mice were purchased from Jackson Laboratory. *Foxn1^lacZ/lacZ^* (Z/Z) and *Foxn1^Cre^* mice were generated by Dr. Manley’s laboratory as described previously and are maintained on a C57Bl6/J background (37, 38). *Lin28a^fl/fl^b^fl/fl^* mice (*Lin28b^tm2.1Gqda^Lin28b^tm2.1Gqda^/J*, stock No. 02395) were purchased from the Jackson Laboratory (39). *R26iLin28a* inducible transgenic mice were originally provided by Eric Moss (Department of Molecular Biology, Rowan University, Stratford, NJ 08084 USA) and transferred from Dr. Jianfu Chen (Department of Genetics, University of Georgia. Current address: Herman Ostrow School of Dentistry of USC) (40). The *Lin28a^fl^* ^/^ *^fl^b^fl/fl^;Foxn1^Cre^* TEC-specific deletion of *Lin28a* and *b* and *R26iLin28a*;Foxn1Cre TEC-specific overexpression of *Lin28a* mice were generated by crossing the *Lin28a^fl/fl^b^fl/fl^* strain or the *R26iLin28a* strain with the *Foxn1^Cre^* mouse strain, respectively. All analyses were performed using littermate animals whenever possible. Data from both male and female mice were combined because no difference was detected based on sex. All mice were maintained in a pathogen-free facility at the University of Georgia. The experiments were approved by the University of Georgia Institutional Animal Care and Use Committee.

### Flow cytometry analysis and antibodies

For thymocyte analysis, freshly isolated thymocytes in suspension (1×10^6^) were used for each sample. Cells were blocked with an anti-CD16/32 (Clone: 93) antibody before staining. For a phenotypic profile of thymocyte subsets, the thymocytes were incubated with anti-CD4 APC-Cy7 (GK1.5), anti-CD8 PE-cy7 (53-6.7), anti-CD44 allophycocyanin (APC) (IM7), and anti-CD25 PerCP (3C7) on ice for 20 min.

For TEC isolation, one thymic lobe (fetal thymi were mixed) was cut into approximately 1 mm^3^ sections and gently washed in 2% FBS RPMI 1640 medium to remove thymocytes. The thymic pieces were digested in 5 ml of collagenase/dispose (1 mg/ml, Roche) plus DNase I (20 ng/mL, Sigma) in 2% FBS RPMI 1640 medium, placed in a 37°C water bath for 60 minutes, and agitated by passing through an 18G needle 8 times and a 25G needle twice. Cells were then filtered by passing through a 70 μm cell strainer.

For TEC phenotypical analysis, the digested cells (1-2 ×10^6^) were incubated with anti-CD45-PE-Cy7 (30-F11), MHCII-PerCp (M5/114.15.2), EpCAM-APC (G8.8), UEA-1-FITC (Vector, CA), and Ly51-PE (6C3) for 20 min. Cells were then placed in a 1% PFA PBS solution for analysis. For LIN28a antibody staining, we followed the Cell Signaling protocol for intracellular antibody staining. Briefly, digested thymic cells (1-2 ×10^7^/ml) were fixed in 4% PFA-PBS and placed in a 37°C water bath for 10 min. The cells were permeabilized by slowly adding 1 ml of ice-cold 90% methanol and incubating for 30 min on ice. The cells were then washed in 2% FBS-PBS buffer and incubated with anti-LIN28a (1:200) and UEA-1-Biotin at RT for 1 hr. The cells were then washed and incubated with the following antibodies: anti-CD45-PE-Cy7, MHCII-PerCp, EpCAM-APC, Ly51-PE, Strepavidin-APC-Cy7, and donkey anti-Rabbit-Alexa-488 (1:800) at RT for 30 min. For BrdU and LIN28a staining together, anti-CD45-PE-Cy7, MHCII-PerCp, EpCAM-PE, UEA-1-biotin following streptavidin-APC-Cy7, BrdU-FITC and LIN28a following donkey anti-Rabbit-Alexa-647 were used.

For TEC sorting, the digested cells were incubated with anti-CD45 APC, EpCAM PE, and MHCII FITC. The CD45^-^Epcan^+^MHCII^+^ cells were sorted as TECs and separated into MHCII^hi^ and MHCII^lo^ subpopulations by MoFlo-DXP cytometry (Dako Cytomation). All antibodies were purchased from Biolegend if not noted (San Diego, CA). Phenotypical analysis was performed with a Cyan ADP Flow Cytometer (Beckman Coulter, Miami, FL). The data were analyzed by Flowjo™ Software (Tree Star, Ashland, OR).

### Immunofluorescence

Primary antibodies included specific polyclonal rabbit anti-LIN28a (A117, Cell Signaling Technology, Beverly, MA; 1:100); specific polyclonal rabbit anti-LIN28b (ProteinTech Group, Inc; Chicago, IL; 1:50) (25, 41); goat anti-mouse FOXN1 (WHN G-20, Santa Cruz Biotechnology; Santa Cruz, CA. 1:200); and rat anti-IA/IE (M5/114.15.2, Biolegend, San Diego, CA 1:200). Secondary antibodies included donkey anti-rabbit IgG-FITC (1:200), donkey anti-goat IgG Alexa-555 (1:800), donkey anti-rat IgG Alexa-555 (1:800); UEA-1-biotin (Vector Laboratories, Brulingame, CA 1:200); and streptavidin Alexa-647 (1:800) (Jackson Immunoresearch Laboratories, West Grove, PA). For FOXN1 staining, the embryos and adult thymi were fixed with 4% paraformaldehyde (PFA) in PBS at 4°C overnight. The samples were dehydrated in gradient ethanol solutions (70, 80, 90, 96, and 100%), embedded in paraffin, and then sectioned at 10 μm. The slides were rinsed in xylene before rehydration through a reversed ethanol series. Antigen retrieval was performed by boiling slides in pH 6, 10 mM sodium citrate buffer for 30 mins. For MHCII staining, embryos and adult thymi embedded in OCT were snap-frozen in liquid nitrogen and then stored at -80°C until sectioning. These samples were cut at 10 μm and fixed in ice-cold acetone for 2 min. All antibodies were diluted in 0.1% BSA+PBS, the primary antibodies were then added to each sample after blockage with 10% donkey serum overnight, and the secondary antibodies or streptavidin were stained for 40 min at RT. Images were obtained using a Zeiss Axioplan2 imaging microscope with an AxioCam HRM and AxioVision Rel 4.5 software (Jena, Germany). Quantitation of fluorescent cells was measured using ImageJ free software.

### BrdU incorporation and Annexin V staining

Each mouse was given a single intraperitoneal injection. with 1 mg of BrdU (Sigma−Aldrich) and hydrated with BrdU-containing water (0.8 mg/ml) for 5 days. One thymic lobe was digested for TEC, and one was ground for thymocyte analysis. For BrdU staining, freshly isolated thymocytes were incubated with anti-CD4-PE-Cy7, CD8-APC-Cy7, CD25-PerCp, and CD44-APC antibodies. The digested cells were incubated with anti-CD45 PE-Cy7, EpCAM APC, MHCII PE, and UEA-1-Biotin following Avidin-APC-Cy7. The surface-stained cells were then fixed and permeabilized in PBS containing 1% paraformaldehyde plus 0.01% Tween 20 for 48 h at 4°C and then incubated with 2 mg/ml DNase I for 15 min in a 37°C water bath. FITC-anti-BrdU Ab (clone 3D4; BD Pharmingen) was used for BrdU staining according to the manufacturer’s instructions. For Annexin V staining, freshly isolated thymocytes (5 X 10^5^) or total digested thymic cells (1 X 10^6^) were incubated with surface antibodies and then incubated with Annexin V FITC and PI in Annexin V binding buffer. The samples were analyzed within 1 hour following the protocol of the Annexin V kit (Biolegend, San Diego, CA).

### RT−PCR and Q-PCR

The fetal thymi and sorted total TECs or MHCII^lo^ and MHCII^hi^ TEC subsets were extracted for mRNA by the RNeasy micro kit (QIAGEN). Reverse transcription PCR was performed with the superscript III system (Invitrogen). Gene expression levels of *Lin28a* (Mm00524077_m1), *Lin28b* (Mm01190673_m1), *Let-7g* (Mm04231484_s1), and *H2-IAb* (00439216_m1) in total TECs or MHCII^lo^ and MHCII^hi^ subsets of TECs were measured by Q-PCR, with a *GAPDH* FAM (Mm99999915_g1) primer/probe used as an endogenous control. All primers/probes were ordered from Applied Biosystems. Q-PCR was performed following the manufacturer’s instructions in a 10 μl volume using the AB 7500 Sequence Detector.

### Sex steroid ablation

Mice were anesthetized using isoflurane anesthesia and then placed in dorsal recumbency. A 0.5-1 cm ventral midline incision was made in the scrotum, and the skin retracted to expose the tunica. The tunica was pierced, and the testes were pushed out sequentially. The testes were raised to expose the underlying blood vessels and tubules. Mouse testes were removed using forceps; cauterization was not needed. All deferential vessels and ducts were replaced back into the tunica. Skin incisions were closed with stainless steel wound clips. All of the excised paired tissues from each animal were retained to verify completeness of removal. Sham castration was performed as described above except for the removal of the testes.

### Statistical analysis

All data were collected in a Microsoft Excel file and analyzed using Prism software (GraphPad Software, Boston, MA 02110) by one-way analysis of variance (ANOVA)-Bonferroni test or Student’s *t* test, *P* value in two-tailed. For statistical analysis, the significance was set at *P* ≤ 0.05.

## Results

### Lin28a and Lin28b are differentially expressed in fetal TECs

**Figure 1:**
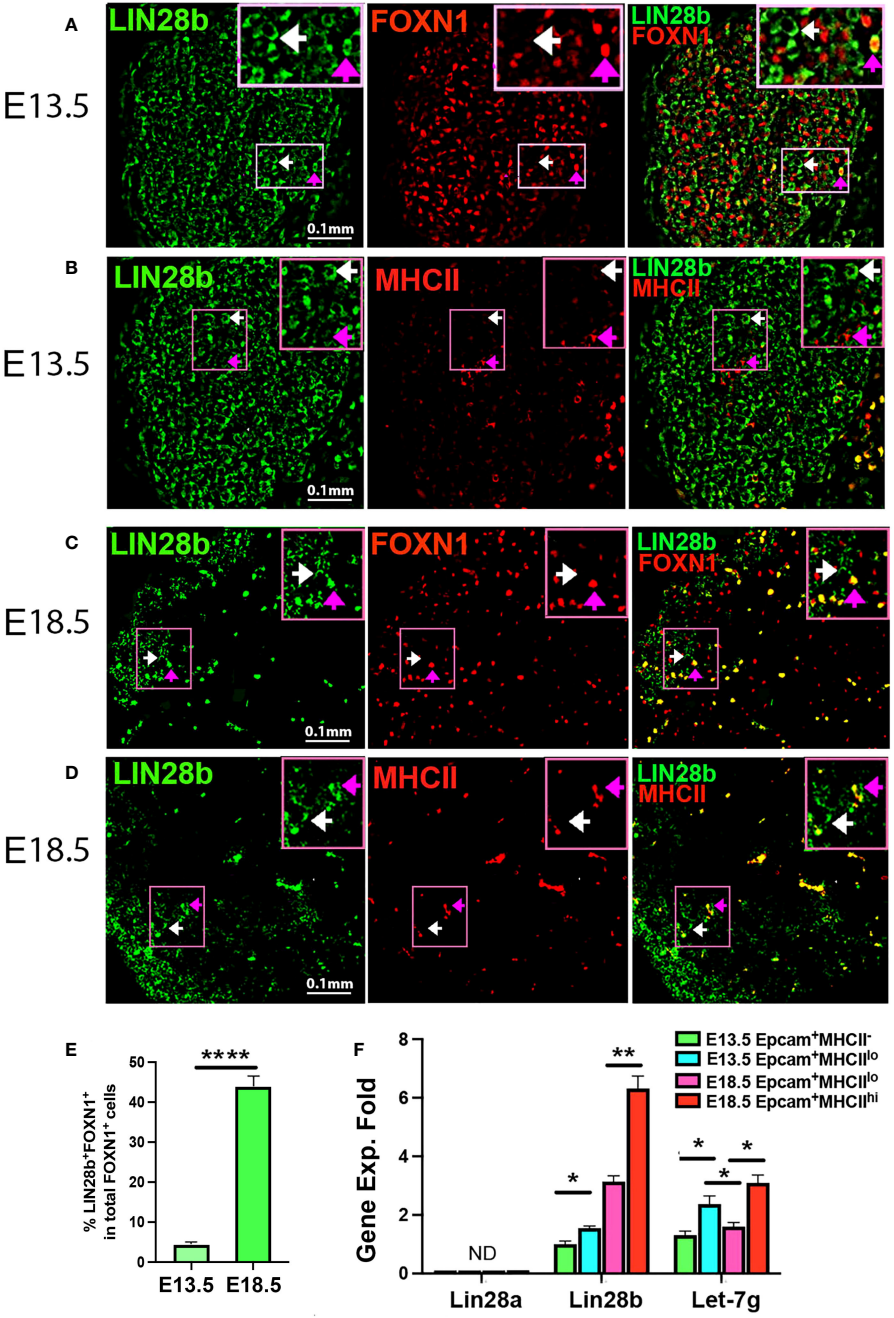
*Lin28a*, *Lin28b*, and *Let-7g* expressed in fetal murine thymi. Immunofluorescence staining of fetal thymic sections. **(A)** 4% PFA-fixed E13.5 thymic sections were stained for LIN28b (green, white arrow shows cytoplasm staining) and FOXN1 (red, pink arrow shows nuclear staining). **(B)** The frozen thymic sections were stained for LIN28b (green) and MHCII (red). **(C)** 4% PFA-fixed E18.5 thymic sections were stained for LIN28b (green) and FOXN1 (red). **(D)** The frozen thymic sections were stained for LIN28b (green) and MHCII (red). The top right shows a digitally enlarged image of the area indicated in panels **(A-D)**. **(E)** Quantitative analysis of LIN28b^+^FOXN1^+^ cells in FOXN1^+^ TECs. **(F)**. Gene expression levels of *Lin28a, Lin28b*, and *Let-7g* were measured in EpCAM^+^MHCII^-^ and EpCAM^+^MCHII^lo^ TECs sorted from E13.5 thymi, as well as in EpCAM^+^MHCII^lo^ and EpCAM^+^MHCII^hi^ TECs sorted from E18.5 thymi. The mRNA level of *Lin28b* in EpCAM^+^MHCII^-^ TECs at E13.5 was used as a reference with a value of 1, and the mRNA levels of other genes were normalized accordingly and expressed as fold changes. Data are representative of two individual experiments and at least 3-5 samples per time point. Student’s *t* test **(E, F)** results between EpCAM^+^ MCHII^-^ or MHCII^lo^ and MHCII^hi^ TEC subsets: *P <0.05, **P <0.01, ****P<0.0001, Scale bar = 0.1 mm. ND, Not detected.

To assess LIN28 in the fetal thymus, we first performed immunofluorescence (IF) on E13.5 and E18.5 thymi from C57BL6/J WT mice using LIN28a and LIN28bspecific antibodies. No LIN28a signal was detected in the E13.5 and E18.5 thymi (**Supplementary Figures 1A, B**). At E13.5, LIN28b was detected in the cytoplasm of both FOXN1^-^negative cells (FOXN1^-^) (**Figure 1A**, white arrows) and in a minor subset of FOXN1-positive (FOXN1^+^) TECs (**Figure 1A**, pink arrows). A small number of MHCII^+^ K14^+^ early medullary TECs (mTECs) were also detected in the center of the thymus but were found to be LIN28b negative (**Figure 1B**, **Supplementary Figures 1C, D**). At E18.5, LIN28b was found to be expressed in a subset of TECs in both the cortex and medulla, colocalizing with FOXN1 and MHCII (**Figures 1C, D** **;** pink arrows). Quantitative analysis showed the frequency of LIN28b^+^ cells increased in total FOXN1^+^ cells (**Figure 1E** **).** Some LIN28b^lo^ FOXN1^-^ cells were also detected in the outer area but were absent from the central area of the thymi, which might represent early developing thymocytes (**Figures 1C, D** **;** white arrows).

To further investigate expression in fetal TECs, we analyzed the gene expression of *Lin28*a and *b* and *Let-7g* in fetal TEC subsets sorted based on EpCAM and MHCII expression at E13.5 and E18.5. At E13.5, all TECs are MHCII negative or low (**Supplementary Figures 2A-D**). *Lin28a* was not detected or was present at very low levels in all fetal TEC subsets. *Lin28b* was correlated with the expression of MHCII, increasing significantly in MHCII^lo^ compared to MHCII^-^ TEC at E13.5, increasing further at E18.5 in MHCII^lo^ TEC, and increasing two-fold higher in MHCII^hi^ than in MHCII^lo^ at E18.5 (**Figure 1F**). *Let-7g* expression also correlated with MHCII expression and was higher in MHCII^lo^ TECs than MHCII^-^ TECs at E13.5 and higher in MHCII^hi^ than MHCII^lo^ TECs at E18.5. However, at E18.5, expression declined in MHCII^lo^ TECs relative to that at E13.5 (**Figure 1F**), suggesting more complex regulation. These results indicated that *Lin28b* and *Let7g* but not *Lin28a* may be involved in TEC differentiation during fetal thymic development.

### Lin28b and Let-7g are anticorrelated in TEC subsets in the postnatal thymus

**Figure 2:**
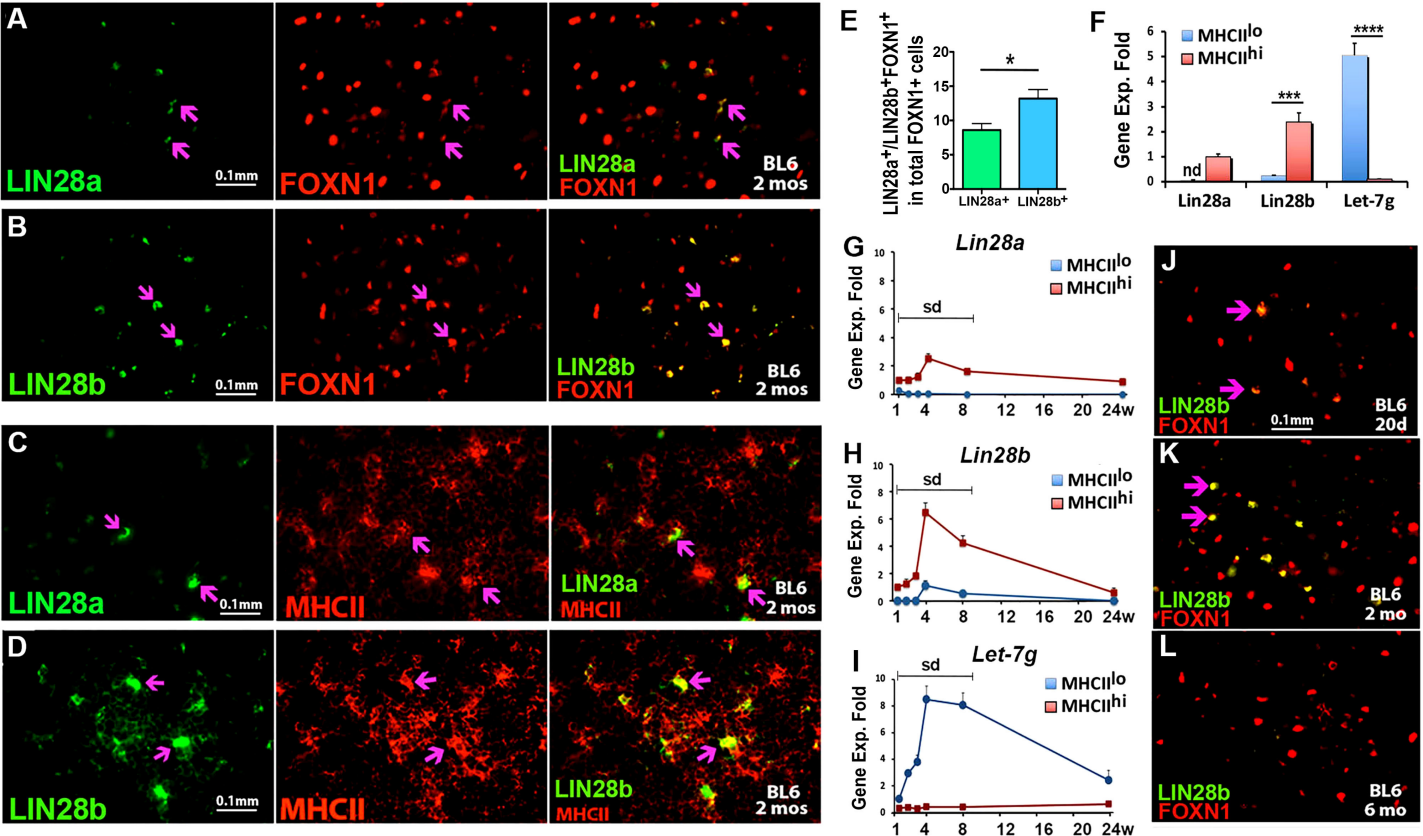
*Lin28a*, *Lin28b* and *Let-7g are* expressed in postnatal murine thymi. Immunofluorescence staining of thymic sections from BL6 WT mice at 2 months of age **(A-D)**. **(A, B)**. 4% PFA-fixed thymic sections were stained for LIN28a (A, green) or LIN28b (B, green) with FOXN1 (red). **(C, D).** The frozen thymic sections were stained for anti-LIN28a (C, green), anti-LIN28b (D, green), and anti-MHCII (red). **(E)** Quantitative analysis showed the frequency of LIN28a^+^ and LIN28b^+^ cells in total FOXN1^+^ TECs. **(F)** Gene expression levels of *Lin28a, Lin28b* and *Let-7g* were measured by qPCR in MHCII^lo^ and MHCII^hi^ TEC subsets sorted from 2-month-old murine thymi. The mRNA level of *Lin28a* in MHCII^hi^ subsets was used as a reference with a value of 1, and the mRNA levels of *Lin28b* and *Let-7g* were normalized accordingly and expressed as fold changes. **(G-I).** Gene expression levels of *Lin28a, Lin28b*, and *Let-7g* were measured by qPCR in MHCII^lo^ and MHCII^hi^ TEC subsets sorted from 1-, 2-, 3-, 4-, 8- and 24-week-old thymi. The mRNA levels of *Lin28a* and *Lin28b* in MHCII^hi^ TECs and *Let-7g* in MHCII^lo^ TECs at week 1 were used as references with a value of 1, and the mRNA levels of *Lin28a, Lin28b*, and *Let-7g* at other time points were normalized accordingly and expressed as fold changes. Each time point represents at least three to five individuals. **(J-L).** Immunofluorescence staining, 4% PFA-fixed thymic sections from BL6 white mice at 20 days **(J)**, 2 months **(K)**, and 6 months **(L)** of age were stained for LIN28b (green) and FOXN1 (red) antibodies. The double-positive staining is shown in yellow (purple arrows). One-way ANOVA **(F-H)** results are shown between MHCII^lo^ and MHCII^hi^ TEC subsets: *P <0.05, ***P <0.001, ****P <0.0001. Bars indicate means ± SEMs. Nd, not detected; Sd, significant difference. Scale bar = 0.1 mm.

To understand *Lin28* and *Let-7* expression in the postnatal thymus, we first assessed LIN28a and LIN28b at the protein level in postnatal TECs. Sections from C57BL6/J WT thymi obtained at 2 months of age were stained along with FOXN1 and MHCII. Both LIN28a and LIN28b in the medulla colocalized with both FOXN1 (**Figures 2A, B**) and MHCII (**Figures 2C, D**), indicating that they are each expressed in a subset of mTECs at this stage. Quantitative analysis showed the frequency of LIN28b^+^ cells in total FOXN1^+^ cells was higher than LIN28a^+^ cells (**Figure 2E**). We further investigated the gene expression of *Lin28a*, *Lin28b*, and *Let-7g* in sorted MHCII^lo^ and MHCII^hi^ TEC subsets from 2-month-old WT mice (**Figure 2F**). Both *Lin28a* and *Lin28b* were preferentially expressed in MHCII^hi^ TECs with little or no expression in MHCII^lo^ TECs. *Lin28b* expression was 2.8-fold higher than that of *Lin28a*. Conversely, *Let-7g* was almost exclusively expressed in MHCII^lo^ TECs (**Figure 2F**). This opposing expression profile is consistent with the known reciprocal regulation pattern for *Lin28* and *Let-7* in other tissues. These patterns for *Lin28a* and *Lin28b* are at least broadly consistent with *Let-7g* expression. However, the *Let-7g* pattern is the opposite of that observed at E18.5, where *Let-7g* was in both MHCII subsets but higher in MHCII^hi^ (**Figure 1F**), which suggests complex regulation of this miRNA gene.

### Temporal dynamics of Lin28a, Lin28b, and Let-7g expression with aging

We next traced the temporal dynamics of *Lin28a, Lin28b*, and *Let-7g* expression in postnatal MHCII^lo^ and MHCII^hi^ TEC subsets. The expression of both *Lin28a* and *Lin28b* gradually increased from 1 week, peaked at 1 month, and then gradually declined at 2 and 6 months in MHCII^hi^ TECs; a parallel pattern was found at a much lower level for *Lin28b* in MHCII^lo^ TECs (**Figures 2G, H**). *Let-7g* showed a similar temporal profile but in MHCII^lo^ TECs, with very low levels in MHCII^hi^ TECs (**Figure 2I**). Consistent with these gene expression kinetics, IF staining showed that LIN28b was present at a low level in a subset of FOXN1^+^ mTECs at day 20 (**Figure 2J**), increasing in cell number and intensity per cell at 2 months (**Figure 2K**), then dramatically declined or was undetectable at 6 months (**Figure 2L**). These data indicate that the expression of *Lin28a*, *Lin28b*, and *Let-7g* is correlated with both thymic growth and the early stages of age-associated involution.

### Specific deletion of Lin28a and Lin28b in TECs caused a reduction in TECs and generated fewer thymocytes in the postnatal thymus

**Figure 3:**
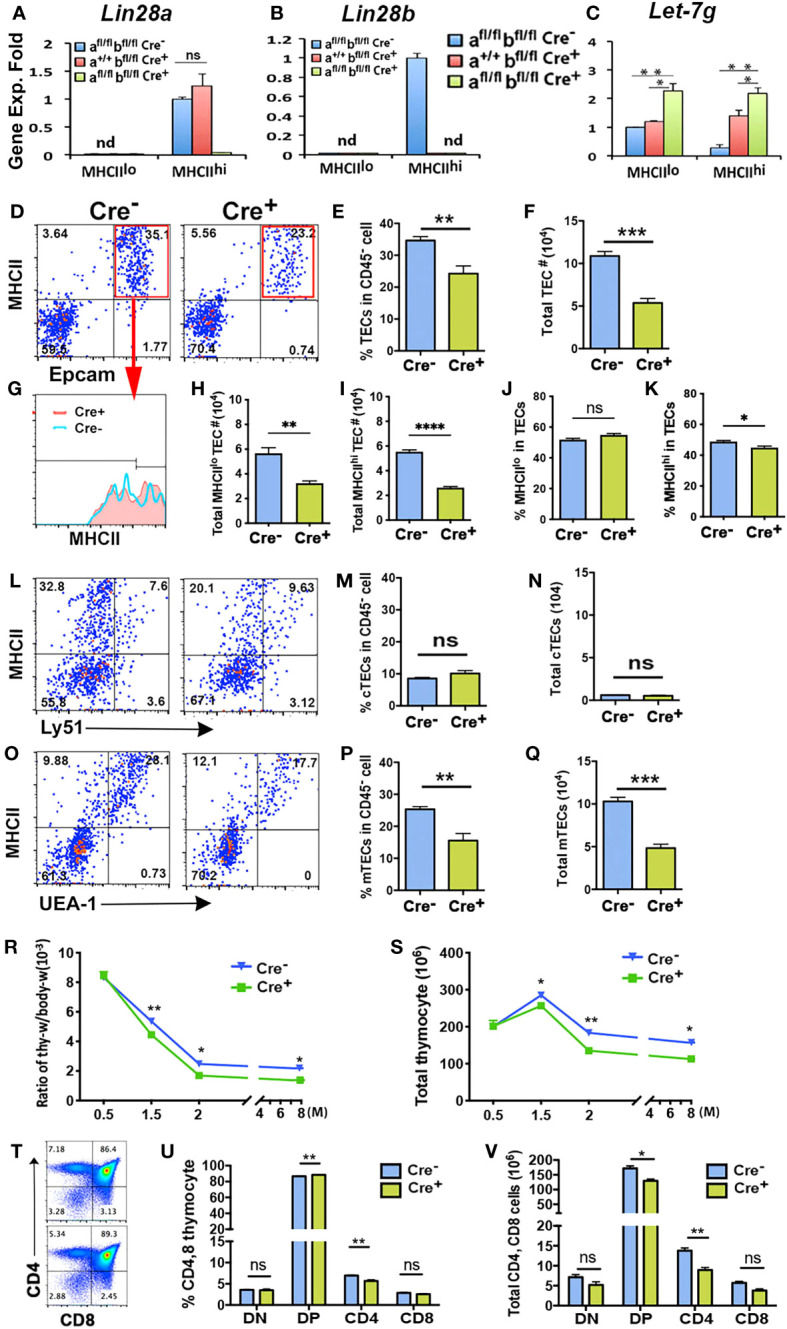
Specific deletion of *Lin28a* and *Lin28b* in TECs caused a reduction of TECs and generated fewer thymocytes in the adult murine thymus. **(A-C)**. Gene expression of *Lin28a* **(A)**, *Lin28b* **(B)**, and *Let-7g* **(C)** was measured in MHCII^lo^ and MHCII^hi^ TECs sorted from *Lin28a^fl/fl^b^fl/fl^, Foxn1Cre^-^* (Cre^-^, n= 4), *Lin28a^+/fl^b^fl/fl^,Foxn1Cre^+^* (n=3) and *Lin28a^fl/fl^b^fl/fl^,Foxn1Cre^+^* (Cre^+^, n=4) mice. Data are representative of two individual experiments. The gene expression levels of *Lin28a* **(A)** and *Lin28b* **(B)** in *Lin28a^fl/fl^b^fl/fl^, Foxn1Cre^-^* MHCII^hi^ TECs and *Let-7g* **(C)** in *Lin28a^fl/fl^b^fl/fl^, Foxn1Cre^-^* MHCII^lo^ TECs were used as controls with a value of 1. **(D)**. Flow cytometry analysis showed the profile of EpCAM and MHCII staining in total CD45^-^ cells of Cre^-^ and Cre^+^ mice and the gate of EpCAM^+^MHCII^+^ for TECs. **(E, F)**. Histogram showing the percentage of TECs in CD45^-^ cells **(E)** and the total number of TECs **(F)**. **(G)**. Histogram showing MHCII expression on gated TECs in Cre^-^ and Cre^+^ mice. **(H, I)**. Total number of MHCII^lo^ **(H)** and MHCII^hi^ **(I)** TECs. **(J, K)**. Percentage of MHCII^lo^ **(J)** and MHCII^hi^ **(K)** TEC subsets in gated total MHCII^+^EpCAM^+^ TECs. **(L)**. Representative profile of MHCII and Ly51 showed cTECs in CD45^-^ cells. **(M, N)**. Percentage of cTECs) **(M)** in CD45^-^ cells and total number of cTECs **(N)**. **(O)**. Representative profile of MHCII and UEA-1 showed TECs) in CD45^-^ cells. **(P, Q)**. Percentage of mTECs **(P)** in CD45^-^ cells and total number of mTECs **(Q)**. **(R)**. Ratio of thymus weight/body weight at the indicated months of age. **(S)**. Total thymocyte number at the indicated months of age. **(T)**. Representative expression of CD4 and CD8 on total thymocytes. **(U)**. Percentage of CD4 and CD8 thymocytes. **(V)**. Total number of CD4 and CD8 thymocytes. Each time point represents at least three to five individuals. Student’s *t* test results between Cre^-^ and Cre^+^ mice: *P <0.05, **P <0.01 ,***P <0.001,****P < 0.0001.. Bars indicate means ± SEMs.

*Lin28a* and *Lin28b* single and double null mutants have a variety of defects that could confound analysis of their function in TECs (20, 21); therefore, we generated TEC-specific deletion of *Lin28a* and *Lin28b* using *Foxn1^+/Cre^* (38). Since similar genetic and phenotypic analysis results were shown at multiple time points, we show here representative results from 2-month-old mice. Q-PCR analysis showed that at 2 months, both *Lin28a* and *Lin28b* transcripts were undetectable in *Lin28a^fl/fl^b^fl/fl^;Foxn1^Cre/+^* double mutant TECs and that only *Lin28a* was detected in the MHCII^hi^ TECs from *Lin28a^+/+^b^fl/fl^;Foxn1^Cre/+^* single mutants (**Figures 3A, B** **)**. Specificity of LIN28a and LIN28b antibody staining on the thymic sections from *Lin28a^fl/fl^b^fl/fl^*;Foxn1^+/+^ control and *Lin28a^fl/fl^b^fl/fl^*;Foxn1^Cre/+^ double knockout adult mice confirmed the specific deletion of *Lin28a* and *b in TECs* **(** **Supplementary Figures 3A–D** **)**. Deletion of *Lin28b* alone or of both *Lin28a* and *Lin28b* was associated with increased *Let-7g* expression in MHCII^hi^ TECs and even showed a 1.3-fold increase in MHCII^lo^ TECs (**Figure 3C** **)**, despite overall low expression of both *Lin28a* and *Lin28b* in these cells. These data support a negative regulatory relationship between *Lin28* and *Let-7g* expression in MHCII^hi^ TECs.

We next assessed the role of *Lin28* and *Let-7g* in TEC differentiation and MHCII expression in 2-month-old double mutant mice. TEC-specific deletion of both *Lin28a* and *Lin28b* caused a significant reduction in both the percentage of TECs within total CD45^-^ stromal cells and in total TEC number (**Figures 3D–F**). Reductions were similar in both MHCII^lo^ and MHCII^hi^ TEC numbers, with a slight decline in the frequency of MHCII^hi^ TECs (**Figures 3G–K**). Analysis of cortical (cTEC) and mTEC populations showed that this reduction was entirely in mTECs in the *Lin28a^fl/fl^b^fl/fl^ Foxn1^Cre/+^* double mutants (**Figures 3L–Q**). These TEC defects caused a significant reduction in thymus size (**Figure 3R**) and in total thymocyte number (**Figure 3S**) starting at 6 weeks of age. The overall decline in thymocyte numbers was reflected in small but significant declines in DP and CD4SP numbers (**Figures 3T–V**). These results suggest that *Lin28a* and *Lin28b* impact thymocyte differentiation indirectly, primarily by regulating the differentiation and/or proliferation of MHCII^hi^ mTECs in the postnatal thymus.

### Lin28a overexpression in TECs increased the total TEC number and produced more thymocytes

**Figure 4:**
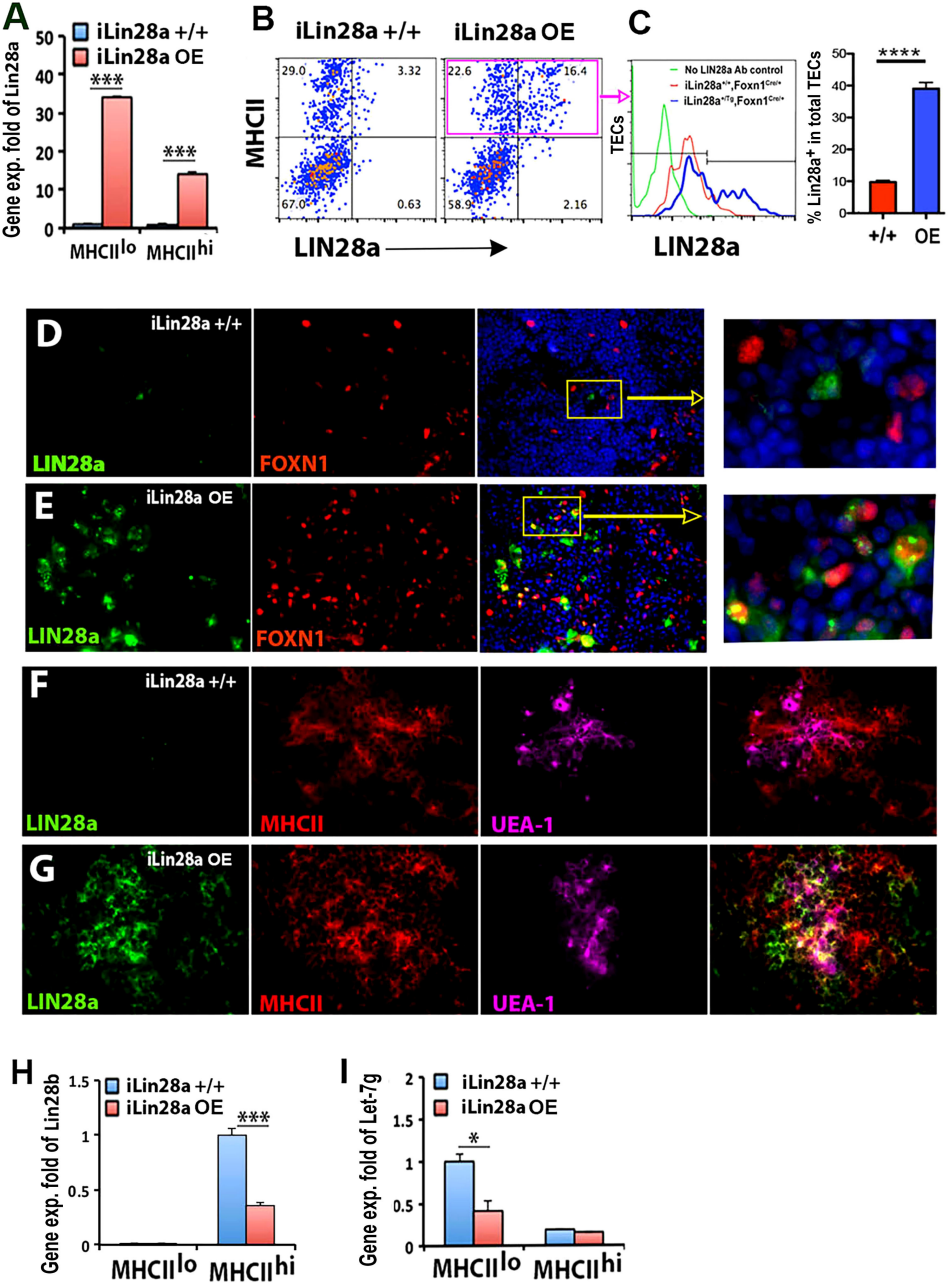
Specific overexpression of LIN28a in TECs in *iLin28a* transgenic adult mice. **(A)** Gene expression of *Lin28a* was measured in MHCII^lo^ and MHCII^hi^ TECs sorted from *iLin28a^+/+^* WT (+/+) and *iLin28a OE* transgenic (OE) mice at 6 weeks of age. Data are representative of two individual experiments (+/+: n= 4; OE: n=5). **(B)** Flow cytometry intracellular staining analysis. Representative profiles of LIN28a and MHCII staining in total CD45^-^ cells. **(C)** The histogram shows LIN28a overlapping expression on gate MHCII^+^ cells, and the summary data of LIN28a percentage are shown on the right. **(D-G)** Immunofluorescence staining, 4% PFA-fixed postnatal day 21 thymic sections, the thymi of +/+ **(D)** and OE **(E)** mice were stained for LIN28a (green), FOXN1 (red) antibodies, and DAPI (blue). The frozen thymic sections of +/+ **(F)** and OE **(G)** mice were stained for anti-LIN28a (green) and MHCII (red) antibodies and UEA-1 (purple). **(H, I)** Gene expression of *Lin28b* **(H)** and *Let-7g* **(I)** was measured in MHCII^lo^ and MHCII^hi^ TECs sorted from *+/+* **(H)** and *OE* **(I)** mice at 6 weeks of age. Data are representative of three individual experiments. (+/+: n=7, OE: =5) Student’s *t* test **(A, C, H, I)** results between +/+ and OE mice: *P <0.05, ***P <0.001, ****P<0.0001. Bars indicate means ± SEMs. Scale bar = 0.1 mm.

To further test LIN28 activity in TECs, we overexpressed (OE) *Lin28a* by activating the *iLin28a* transgene (*iLin28a-Tg*) (40) specifically in TECs with *Foxn1Cre* (38). Activation of the transgene resulted in a 34-fold increase in *Lin28a* expression in MHCII^lo^ TECs and a 14-fold increase in MHCII^hi^ TECs in 6-week-old *iLin28a^+/Tg^*;*Foxn1^Cre/+^* (*iLin28a* OE) transgenic mice (**Figure 4A** **)**. Flow cytometry analysis was consistent with these data, with a low level of LIN28a signal detected in MHCII^+^ TECs in *iLin28a^+/+^* controls and a strong LIN28a signal in the *iLin28a* OE mice in both MHCII^lo^ and MHCII^hi^ TECs, although LIN28a overexpression was detected only in a subset of TECs (**Figures 4B, C**). Consistent with the mRNA levels and flow cytometry, LIN28a was difficult to detect by IF in the *iLin28a^+/+^* control thymus at 3 weeks of age (**Figures 4D, F**), but LIN28a dramatically increased in *iLin28a* OE mice (**Figures 4E, G**). LIN28a protein in transgenics was found primarily in the cytoplasm but was also present in the nucleus of TECs (**Figure 4E** **, **inset). Interestingly, *Lin28b* expression in MHCII^hi^ subsets was reduced in *iLin28a* OE mice compared to controls (**Figure 4H**), which suggests cross-regulation of these two related genes. Expression of the transgene was associated with a 60% reduction in *Let-7g* in MHCII^lo^ TECs but no significant change in *Let-7g* in MHCII^hi^ TECs (**Figure 4I**), possibly because of the reduction in *Lin28b* expression in these cells.

**Figure 5:**
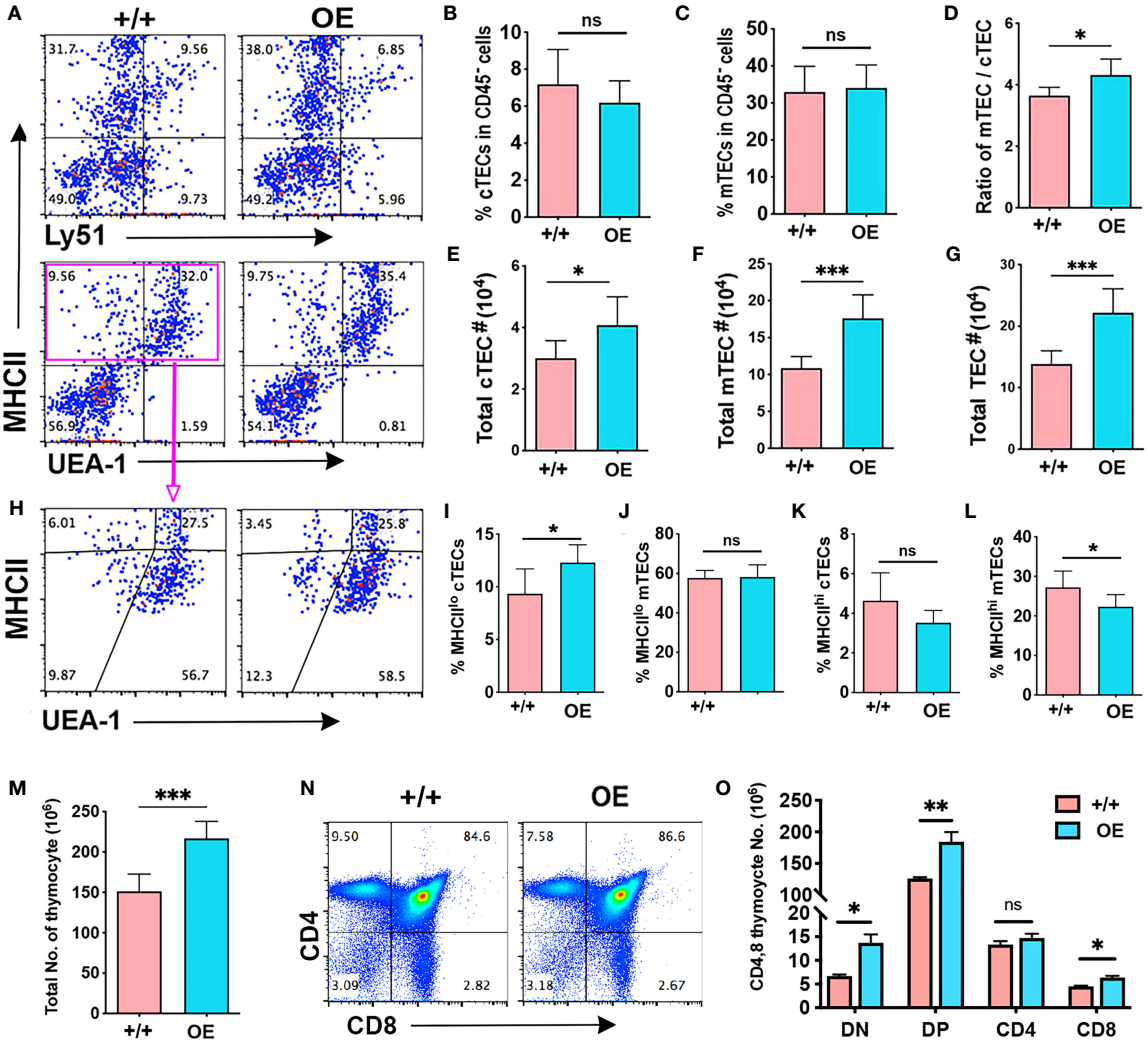
Specific *Lin28a* overexpression in TECs increased the total TEC number and produced more thymocytes. Flow cytometry analysis data. **(A)** Representative expression of MHCII and Ly51 (top) or UEA-1 (bottom) on total CD45^-^ cells. **(B-D)** Histogram showing the percentage of cTECs **(B)** and mTECs **(C)** in total CD45^-^ cells and the ratio of mTECs/cTECs **(D)**. **(E-G)** Total number of cTECs **(E)**, mTECs **(F)** and total TECs **(G)**. **(H)** Representative profiles of MHCII and UEA-1 staining in gated total TECs (EPCAN^+^MHCII^+^). **(I-L)**. **(I-L)** Percentage of MHCII^lo^ cTECs **(I)**, MHCII^lo^ mTEC **(J)**, MHCII^hi^ cTECs **(K)**, and MHCII^hi^ mTEC **(L)**. **(M)** Total number of thymocytes. **(N)** Representative profiles of CD4 and CD8 staining of total thymocytes. **(O)** Total number of CD4 and CD8 subsets of thymocytes. Student test results between +/+ and OE mice: *P <0.05, **P<0.01, ***P <0.001.

Overexpression of *iLin28a* in TECs caused an increase in thymus weight and the ratio of thymus weight to body weight in *iLin28a* OE mice (**Supplementary Figures 4A-D**), although it did not change the frequencies of cTECs and mTECs (**Figures 5A–D**). Increased thymus size was associated with significant increases in the number of both cTECs and mTECs (**Figures 5E–G**). To further analyze TEC phenotypes, we assessed MHCII expression. The frequency of MHCII^lo^ cTECs was increased, and the frequency of MHCII^hi^ mTECs was reduced in *iLin28a* OE mice (**Figures 5H–L**). Consistent with the overall increased size, the numbers of all MHCII subsets were increased, except for MHC^hi^ cTECs (**Supplementary Figures 4E, F**). Overall, the ratio of MHCII^lo^ to MHCII^hi^ cells increased in *iLin28a* OE mice (**Supplementary Figure 4G**).

Consistent with increased thymus size and TEC number, total thymocyte number was significantly increased in *iLin28a* OE mice (**Figure 5M**) with increases in the number of all subsets except for CD4SP cells, which declined in percentage (**Figures 5N, O** **;** **Supplementary Figure 4H**). The decline in CD4SP cell frequency is consistent with the decline in the frequency of MHCII^hi^ mTECs in *iLin28a* OE mice (**Figure 5L**).

### Overexpression of Lin28a promotes the proliferation of MHCII^lo^ TECs

**Figure 6:**
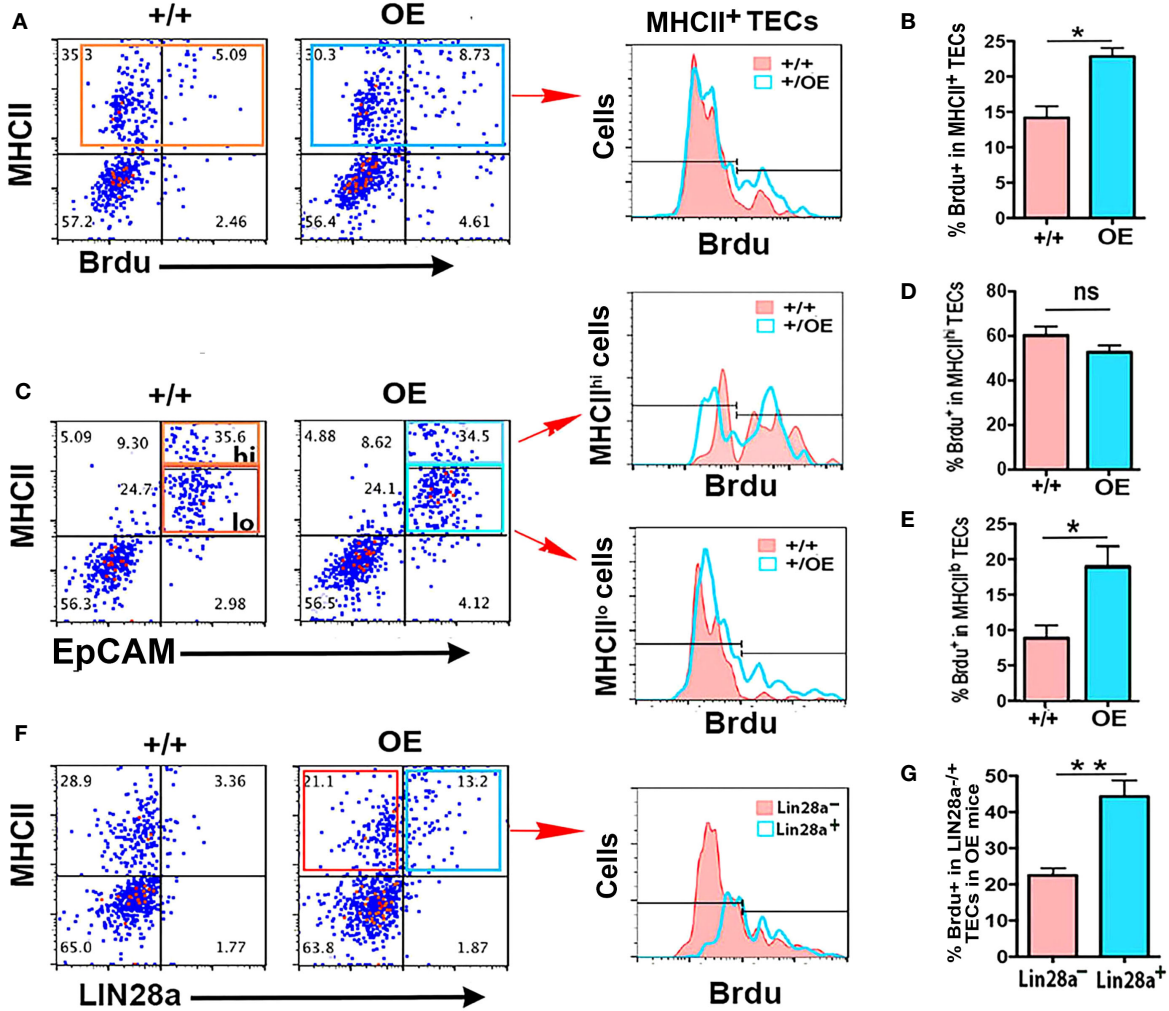
Specific overexpression of Lin28a in TECs increased the proliferation capability of MHCII^lo^ TECs. **(A)** Representative profiles of MHCII and BrdU staining on total CD45^-^ cells in +/+ and OE mice and histograms show overlapping profiles of BrdU staining on gated MHCII^+^ cells. **(B)** Summary of the percentage of BrdU^+^ cells in total TECs. **(C)** Representative profiles of MHCII and EpCAM staining on gated CD45^-^ cells and the gates of MHCII^lo^ and MHCII^hi^ TEC subsets in TECs, and histograms show overlapping profiles of BrdU staining on gated MHCII^hi^ and MHCII^lo^ TECs. **(D)** Percentage of BrdU^+^ cells in MHCII^hi^ TECs. **(E)** Percentage of BrdU^+^ cells in MHCII^lo^ TECs. **(F)** Representative profiles of MHCII and *Lin28*a staining on gated CD45^-^ cells and histograms show overlapping profiles of BrdU staining on gated Lin28a^-^ (red) and Lin28a^+^ (blue) TECs. **(G)** Percentage of BrdU^+^ cells in gated Lin28a^-^ and Lin28a^+^ TECs from OE mice only. Data are representative of two individual experiments (+/+: n= 5; OE: n=5). Data are representative of three individual experiments. Mice aged 6-7 weeks were used. Student’s t test results between +/+ and OE mice: *P <0.05, **P <0.01. Bars indicate means ± SEMs. ns: not significant.

The above results showed that TEC-specific deletion of both *Lin28a* and *Lin28b* caused a selective reduction in MHCII^hi^ mTECs, while overexpression of *Lin28a* expanded all TEC subsets but preferentially expanded MHCII^lo^ TECs. Because *Lin28a* and *Lin28b* are typically expressed in stem/progenitor cells associated with cell proliferation, we hypothesized that *Lin28a* and *Lin28b* promote TEC proliferation in the postnatal thymus, especially in MHCII^lo^ TECs. To test this hypothesis, we measured TEC proliferation in *iLin28a* OE mice at 6 weeks of age. Compared to control mice, in *iLin28a* OE mice, the total number of BrdU^+^ TECs was significantly increased in total TECs (**Figures 6A, B** **)** but was selectively higher in MHCII^lo^ TECs (**Figures 6C–E** **)**. LIN28a^+^ TECs incorporated a significantly higher level of BrdU than LIN28a^-^ TECs, indicating that this is a cell-autonomous effect (**Figures 6F, G**). In addition, the frequency of BrdU^+^ cells did not change in thymocyte subsets (**Supplementary Figure 5A**), and thymocytes and TECs from both mutant and control mice did not show a significant change in apoptosis (**Supplementary Figures 5B-D**). These data suggest that overexpressing *Lin28a* primarily affects TEC subsets by directly or indirectly increasing the proliferation of MHCII^lo^ TECs.

### Downregulation of Foxn1 expression causes a reduction of Lin28b in MHCII^hi^ and Let-7g in MHCII^lo^ TECs

**Figure 7:**
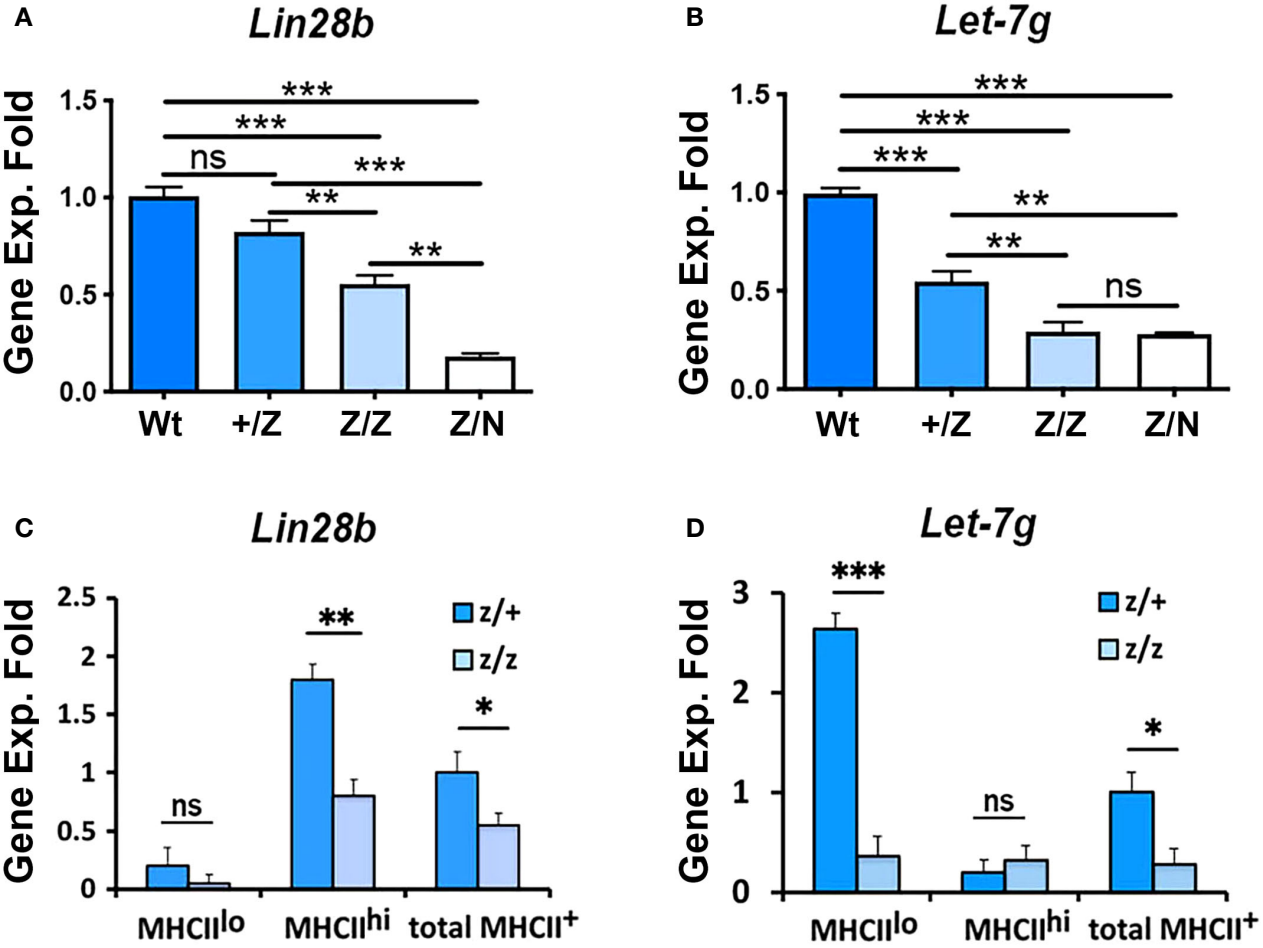
The gene expression of *Lin28b* and *Let-7g* was associated with the Foxn1 expression in the thymus. **(A, B).** Gene expression of *Lin28b* **(A)** and *Let-7g* **(B)** in total TECs from +/+, +/Z, Z/Z, and Z/N thymi of 3-week-old mice. Data are representative of three individual experiments, (+/+: n= 3; +/Z: n=5, Z/Z: n=5, Z/N: n=4). **(C, D).** Gene expression of *Lin28b* **(C)** and *Let-7g* **(D)** in MHCII^lo^ and MHCII^hi^ TEC subsets and total TECs from +/Z and Z/Z thymi. Data are representative of two individual experiments, (+/Z: n=4, Z/Z: n=5). One-way ANOVA **(A, B)** results between each colony of mice; Student’s t-test **(C, D)** results between Z/+ and Z/Z mice: *P <0.05, **P <0.01, ***P <0.001. ns: not significant. Bars indicate means ± SEM.

TEC differentiation and proliferation are also controlled by the transcription factor FOXN1, and *Foxn1* downregulation is considered an initiating event for age-associated thymic involution (37). The dynamic patterns of *Lin28a, Lin28b*, and *Let-7g* across age, the loss of MHCII^hi^ mTECs in *Lin28a and Lin28b* loss-of-function mutants and the selective effect on proliferation of MHCII^lo^ TECs with *Lin28a* OE all mirror aspects of *Foxn1* expression and function (37). To test the relationship between FOXN1 and *Lin28b and Let-7g* in TECs, we generated an allelic series using two alleles of *Foxn1:* the hypomorphic allele *Foxn1^Lacz/LacZ^* (Z/Z) (37) and the null allele *Foxn1^nu^* (42). In *Z/Z* mutants, *Foxn1* expression is downregulated in TECs beginning at P7, resulting in a relative reduction in MHCII^hi^ and an increase in MHCII^lo^ TEC subsets. Combining the Z allele with the nu allele results in a progressive decline in thymus size and TEC phenotypes (37). We first analyzed *Lin28b* and *Let-7g* gene expression in total TECs from *+/+, +/Z, Z/Z* and *Z/N* (*Z/nude*) mice at 3 weeks of age. We found that with the progressive reduction in *Foxn1* expression, both *Lin28b* and *Let-7g* in total TECs were downregulated in a *Foxn1* dose-specific manner (**Figures 7A, B**). Consistent with their wild-type expression patterns, *Lin28b* was reduced in MHCII^hi^ TECs, and *Let-7g* was reduced in MHCII^lo^ TECs in the Z/Z mutants compared to +/Z controls (**Figures 7C, D**). These data indicated that the differential expression of both genes is controlled directly or indirectly by FOXN1.

### Increase in Lin28b expression in sex steroid ablation-induced thymic rebound

**Figure 8:**
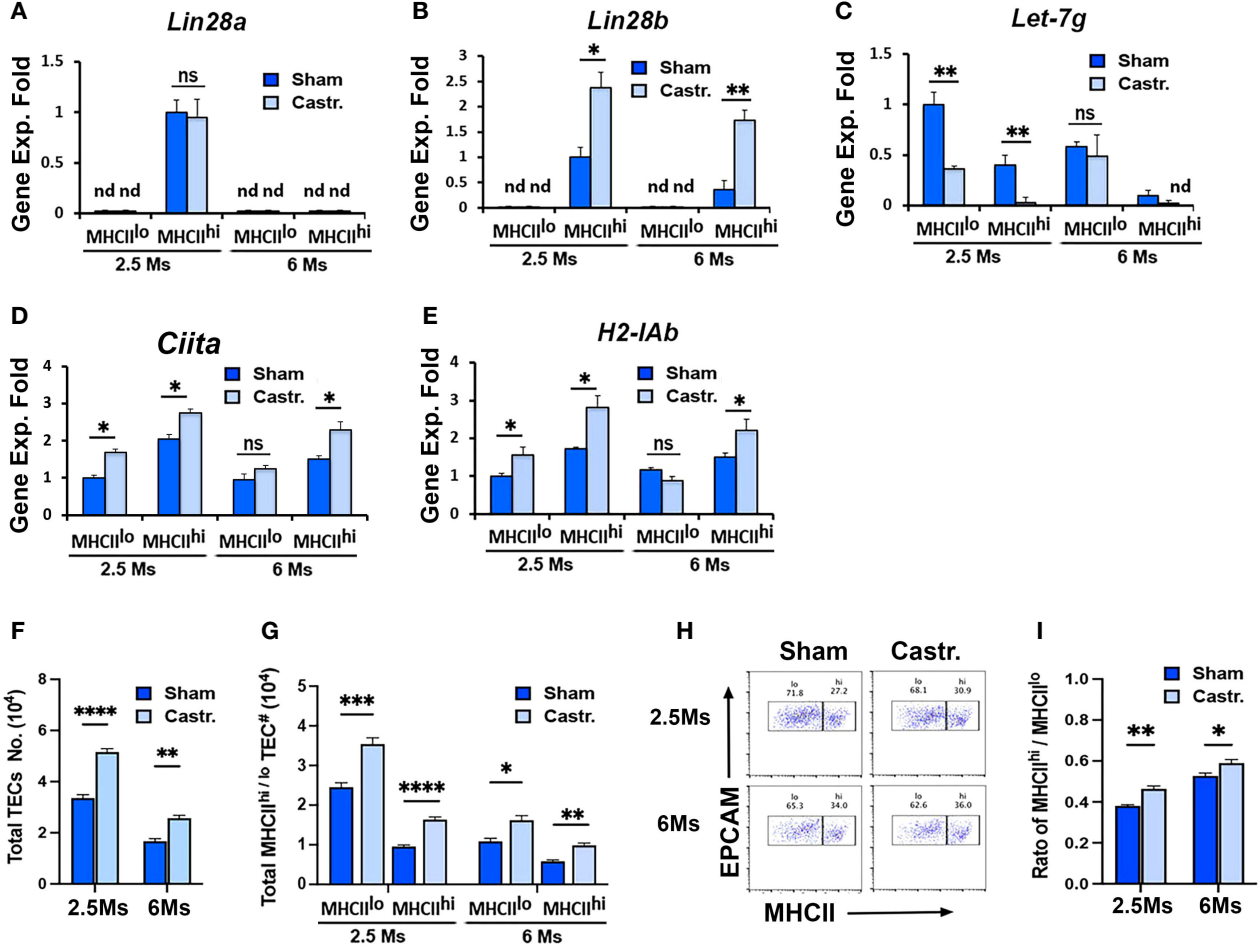
Lin28b increased in sex steroid ablation-induced thymic rebound. 2.5 and 6 months BL6 WT mice underwent castration surgery. Three days later, TECs were isolated, and MHCII^lo^ and MHCII^hi^ TEC subsets were sorted for gene expression analysis and phenotypic analysis. **(A-D)** Gene expression of *Lin28a* **(A)** *, Lin28b* **(B)**, *Let-7g* **(C)**, *Ciita* **(D)**, and *H2-IAb* **(E)** in MHCII^lo^ and MHCII^hi^ TEC subsets in 2.5- and 6-month-old sham and castrated mice. The gene expression levels of *Lin28a and Lin28b* in MHCII^hi^ TECs and *Let-7g* and *H2-IAb* in MHCII^lo^ TECs of 2.5-month sham mice were used as controls with a value of 1. **(F-I)** Flow cytometry analysis data. **(F)** Total number of TECs. **(G)** Total number of MHCII^lo^ and MHCII^hi^ TEC subsets. **(H)** Representative profiles of MHCII and EpCAM staining on gated total TECs. **(I)** Ratio of MHCII^hi^/MHCII^lo^ TECs. Data are representative of two individual experiments, (Sham: n= 5; Castr.: n=6). Student’s t-test results between Sham and Castr mice: *P <0.05, **P <0.01, ***P <0.001, ****P <0.0001. ns: not significant. Bars indicate means ± SEM.

To further investigate the regulation of *Lin28* and *Let-7* relative to thymic involution, we performed sex steroid ablation (SSA)-induced thymic rebound in 2.5- and 6-month-old Wt mice and analyzed gene expression early at day 3.5 after castration. SSA significantly increased *Lin28b* but not *Lin28a* expression specifically in MHCII^hi^ TECs at both 2.5 and 6 months, while expression in MHCII^lo^ TECs remained undetected (**Figures 8A, B** **)**. SSA also caused a reduction in *Let-7g* expression in both MHCII^lo^ and MHCII^hi^ TECs at 2.5 months but no change in either subset at 6 months (**Figure 8C** **)**. Based on *microRNA* search results, we found that the mouse class II transactivator-encoding gene *Ciita* (a critical regulator of *MHCII* gene expression) and several *H2* genes had the predicted target sites for *Let-7g* and some other *Let-7s* (**Table 1**). To test the effect of *Let-7g*, we further measured the changes of these two genes after SSA, and consistent with down-regulation of *Let-7g*, both *Ciita* and *H2-IAb* mRNA were increased in MHCII^lo^ and MHCII^hi^ TECs at 2.5 months, but still increased in MHCII^hi^ TECs at 6 months even without *Let-7g* change (**Figures 8D, E**). These changes in gene expression were associated with an increase in the total number of TECs (**Figure 8F**), including MHCII^lo^ and MHCII^hi^ TECs (**Figure 8G**). Hiweverthe increase was much greater in MHCII^hi^ TECs (**Figure 8H**), causing an increase in the ratio of MHCII^hi^ : MHCII^lo^ (**Figure 8I**). These data suggest that SSA-induced thymic rebound is associated with an increase in *Lin28b* in MHCII^hi^ TECs, with higher frequency of MHCII^hi^ TECs, and increased MHCII gene expression in these cells. The increase in MHCII gene expression in these cells may be caused by reducing the inhibitory effect of *Let-7g* to *Ciita* and *H2* genes.

**Table 1:** Predicted target sites for miRNA *Let-7* in *Ciita* and *H2* genes.

Gene Name	RefSeqID	MicroRNA	Seed Length	Start	Sequence	End	Region	Pvalue	SPMS
**Ciita**	NM_007575	mmu-let-7g*	8	4596	CUGUACAG	4589	3 UTR	0.0274	2
H2-Ab1	NM_207105	mmu-let-7g*	7	1082	CUGUACA	1076	3 UTR	0.0183	2
H2-Ab1	NM_207105	mmu-let-7c-1*	7	1082	CUGUACA	1076	3 UTR	0.0183	1
H2-Eb1	NM_010382	mmu-let-7a*	8	1645	UAUACAAU	1638	3 UTR	0.0119	2
H2-Eb1	NM_010382	mmu-let-7f*	8	1645	UAUACAAU	1638	3 UTR	0.0119	2
H2-Eb1	NM_010382	mmu-miR-7b	7	983	UGGAAGA	977	3 UTR	0.0469	1

*miRNA binding sites within 3’UTR region.

## Discussion

In this study, we linked the expression profiles of *Lin28a, Lin28b*, and *Let-7g* in MHCII^hi^ and MHCII^lo^ TEC subsets to the development and differentiation of TECs during thymic ontogeny and involution. We found that, as in other systems, at murine postnatal stages, *Lin28a, Lin28b*, and *Let-7g* had inversely correlated expression patterns in TECs. Both *Lin28a* and *Lin28b* were more highly expressed in MHCII^hi^ TECs but were expressed at low or undetectable levels in MHCII^lo^ TECs (*Lin28b* was more highly expressed overall than *Lin28a*), while *Let-7g* was expressed at the highest level in MHCII^lo^ TECs but was absent or undetectable in MHCII^hi^ TECs.

Several lines of evidence suggest that *Lin28* and *Let-7* expression and function in TECs are downstream of FOXN1 and are consistent with mediating some of the effects of *Foxn1* downregulation that is thought to precipitate age-related involution. *Lin28a, Lin28b*, and *Let-7g* expression all peak at 1 month of age in their respective cell types and then decline with aging starting at 3 months, similar to *Foxn1* (37). Loss of *Lin28b* specifically in TECs (which causes an increase in *Let-7g*) resulted in a smaller thymus with selective loss of MHCII^hi^ mTECs, while overexpression of *Lin28a* (which causes a loss of *Let-7g*) causes a larger thymus due to selectively increased proliferation in MHCII^lo^ TECs. Both phenotypes are consistent with known roles for *Foxn1* in promoting TEC differentiation and selectively impacting the proliferation of MHCII^lo^ TECs (37). Furthermore, we demonstrated a dose-dependent expression relationship between *Foxn1* expression and both *Lin28b* and *Let-7g* expression, consistent with *Foxn1* acting upstream of both genes. Taken together, these data suggest that at least some functions of *Foxn1* are mediated by its regulation of *Lin28 and Let-7*, both in the steady-state thymus and during involution.

*Lin28a* and *Lin28b* have redundant roles in other cell types at both fetal and adult stages (21, 25, 26). Our data support both unique and redundant functions for *Lin28a* and *Lin28b* in TECs. While only *Lin28b* is expressed at fetal stages, both genes are expressed in postnatal TECs, with similar patterns of expression at least in bulk analysis: higher in MHCII^hi^ than MHCII^lo^ TECs, peaking at 1 month and then declining with age. Comparison of the *Lin28b* single knockout (KO) and *Lin28a* and *Lin28b* double KO phenotypes indicates a redundant function in suppressing *Let-7g* expression, even in MHCII^lo^ TECs where neither gene has much expression. However, their expression patterns do not directly overlap, and immunofluorescence analysis shows that LIN28a and b proteins are expressed in distinct subsets of TECs, with LIN28a protein tending to correlate with lower FOXN1 levels and LIN28b correlating with higher levels of FOXN1. These data suggest that both genes mediate their effects by acting in different subsets of TECs, and while both may act similarly to suppress *Let-7g*, the effects could differ in detail. Their impacts could also differ if *Let-7g* regulates different sets of target genes in different TEC subsets. Regarding the inhibitory effect of LIN28 on *Let-7g*, while the gene expression of *Lin28* and *Let-7g* showed opposite patterns in MHCII^hi^ and MHCII^lo^ TECs during postnatal thymic ontogeny, there were instances where *Let-7g* expression did not follow the changes in *Lin28* expression in TECs. For example, *Lin28b* showed a positive correlation with *Let-7g* expression in E13.5 and E18.5 TECs (**Figure 1F**); specific deletion of *Lin28a* and *b* caused a 1.3-fold increase in MHCII^lo^ TECs (**Figure 3C** **)**, despite overall low expression of both *Lin28a* and *Lin28b* in these cells; overexpression of *Lin28a* did not lead to *Let-7g* reduction in MHCII^hi^ TECs (**Figures 4H, I**); and the rebound of *Lin28b* reduced *Let-7g* expression in MHCII^lo^ TECs in 2.5-month-old but not in 6-month-old SSA mice (**Figure 6G**). These findings suggest that *Let-7g* has additional regulatory inputs beyond LIN28 in TECs (28, 43), which would be a potentially fruitful avenue for future study. The lack of a change in *Let-7g* expression at 6 months combined with the increased expression of *Lin28b* suggests that increases in *H2-IAb* expression correlated specifically with increased *Lin28b* and may be independent of *Let-7g*,

Another important point is that the preferential expression of *Let-7g* in MHCII^lo^ TECs, which are typically considered to represent less mature TECs, is the opposite of what would be predicted from its expression in the most differentiated cells in other cell types. The high expression of *Let-7g* in MHCII^lo^ TECs thus raises the possibility that this expression is due to a subset of MHCII^lo^ postnatal TECs called post-Aire TECs, which represent a senescent, post-proliferative TEC subset that is derived from mature AIRE^+^ mTEC (44, 45). In this case, expression could potentially drive cells into the post-AIRE state. Alternatively, *Let-7g* could have a different pattern and function in TECs compared to other cell types. It may suppress the expression of a critical differentiation marker MHCII in less mature TECs, with upregulation of *Lin28* during TEC differentiation acting to suppress *Let-7g* and thus allow upregulation of MHCII. Supporting this possibility, several *H2* genes and the class II transactivator encoding gene *Ciita*, a critical regulator of *MHCII* gene expression, have *Let-7g* binding sites in their 3’UTRs (**Table 1**). Notably, the expression of *Ciita* and *H2-IAb* were consistent with *Let-7g* down-regulation in 2.5-month SSA mice (**Figures 8D, E**), suggesting that *Lin28* promotes MHCII expression by reducing the inhibitory influence of *Let-7g* on *Ciita* and *H2* genes.

The data from *Foxn1^Lacz^* mutants showed a *Foxn1* dose-dependent downregulation of both *Let-7g* and *Lin28b* expression in TECs, suggesting that expression of both genes requires FOXN1 at some level. This regulation could be direct or indirect, and other regulators are clearly needed to restrict expression to specific TEC subsets. Indeed, mTECs have been implicated in the regulation of *Let-7* levels in developing natural killer T cells in the thymus (46), supporting that TECs can indirectly regulate *Let-7*. Our own data show that the same signals (RA, vitamin D3) regulate *Let-7g* levels in thymic progenitor B cells and depend on *Foxn1* levels (28). Collectively, our data are consistent with a model in which during postnatal TEC development and differentiation, *Let-7g* is broadly upregulated in TECs by increasing FOXN1 levels, while *Lin28b* is upregulated in a subset of TECs due to both FOXN1 and an unidentified regulator(s). In those cells, LIN28b blocks *Let-7g*, allowing for increased MHCII expression and driving the maturation of TECs to MHCII^hi^ cells. With the downregulation of *Foxn1* during aging, both *Let-7g* and *Lin28b* expression decline, but the reduction in *Lin28b* offsets this *Let-7g* downregulation and results in increased inhibition of MCHII expression, which results in long-term declines in MHCII expression during aging and involution.

## Data availability statement

The original contributions presented in the study are included in the article/**Supplementary Material**. Further inquiries can be directed to the corresponding author.

## Ethics statement

The animal study was approved by University of Georgia Institutional Animal Care and Use Committee. The study was conducted in accordance with the local legislation and institutional requirements.

## Author contributions

SX: Conceptualization, Data curation, Formal Analysis, Investigation, Methodology, Writing – review & editing – original draft. WZ: Investigation, Methodology, writing-review & editing. JL: Investigation, Methodology, writing-review & editing. NM: Funding acquisition, Resources, Supervision, Writing – review & editing, Investigation.

## Supplementary material

Supplementary Figure 1 Immunofluorescence staining of fetal thymic sections. **(A, B).** 4% PFA-fixed E13.5 (A) and E18.5 (B) thymic sections were stained by FOXN1 (red) and LIN28a (green, no positive signaling was detected on thymi). **(C).** Frozen E13.5 thymic sections were stained for LIN28b (green), MHCII (red), and K14 (pink). **(D).** The digitally enlarged images of the area indicated in C. Scale bar = 0.1 mm.

Supplementary Figure 2 TECs sorted from fetal and postnatal thymi. **(A-D).** The gate settings for TEC sorting (left). Representative profiles of EpCAM and MHCII staining and gates of EpCAM^+^ MHCII^-^, EpCAM^+^MHCII^lo^, and/or EpCAM^+^MHCII^hi^ TCE subsets in the gated CD45^-^ cells from thymi of fetal E13.5 **(A)**, E18.5 **(B)**, postnatal 30 days **(C)**, and 6 months **(D)**.

Supplementary Figure 3 Immunofluorescence staining of *Lin28* ^a^ and *b* double KO thymic sections. **(A, B).** 4% PFA fixed *Lin28a^fl/fl^b^fl/fl^;Foxn1^+/+^* control (2 month-old) thymic sections were stained by FOXN1 (red) and LIN28a **(A)** and LIN28b **(B)** (green). **(C, D).** 4% PFA fixed *Lin28a^fl/fl^b^fl/fl^;Foxn1^Cre/+^* double KO (2 month-old) thymic sections were stained by FOXN1 (red) and LIN28a **(C)** and LIN28b **(D)** (green, no positive signaling was detected on thymi). Scale bar = 0.1mm.

Supplementary Figure 4 TEC-specific overexpression of Lin28a caused an increase in thymus size in postnatal adult mice**. (A).** Image of thymi from 6-7-week-old *iLin28a* ^+/+^ Wt (+/+) and *iLin28a* OE transgenic (OE) mice. **(B).** Mouse body weights of +/+ and OE mice. **(C).** Thymus weights of +/+ and OE mice. **(D)** Ratio of thymus weight vs body weight of +/+ and OE mice. **(E).** Total number of MHCII^lo^ TECs, including MHCII^lo^ cTECs and mTECs. **(F).** Total number of MHCII^hi^ TECs, including MHCII^hi^ cTECs and mTECs. **(G).** Ratio of MHCII^lo^ TECs vs MHCII^hi^ TECs. **(H).** Percentage of CD4 and CD8 thymocytes in total cells. Student’s t test **(B-D)** results between +/+ and OE mice: *P <0.05, **P <0.01, ***P <0.001, ****P <0.0001. ns: not significant.

Supplementary Figure 5 BrdU and Annexin V staining of cells from TEC-specific overexpression of Lin28a in postnatal adult mice**. (A).** Percentage of BrdU^+^ cells in CD4 and CD8 thymocytes in +/+ and OE mice. **(B).** Percentage of Ann-V^+^ cells in CD4 and CD8 thymocyte in +/+ and OE mice. **(C).** Total Ann-V^+^ CD4 and CD8 thymocytes in +/+ and OE mice. **(D).** Percentage of Ann-V^+^ cells in total TECs in +/+ and OE mice. Data are representative of two individual experiments, (+/+, n=3, OE, n=4). Student’s t test (B-D) results between +/+ and OE mice:. ns: not significant.
