# Supplementary figures and images for: Lin28 regulates thymic growth and involution and correlates with MHCII expression in thymic epithelial cells
Source: Front Immunol. 2023 Oct 6;14:1261081. doi: 10.3389/fimmu.2023.1261081 (PMC10588642; doi:10.3389/fimmu.2023.1261081)

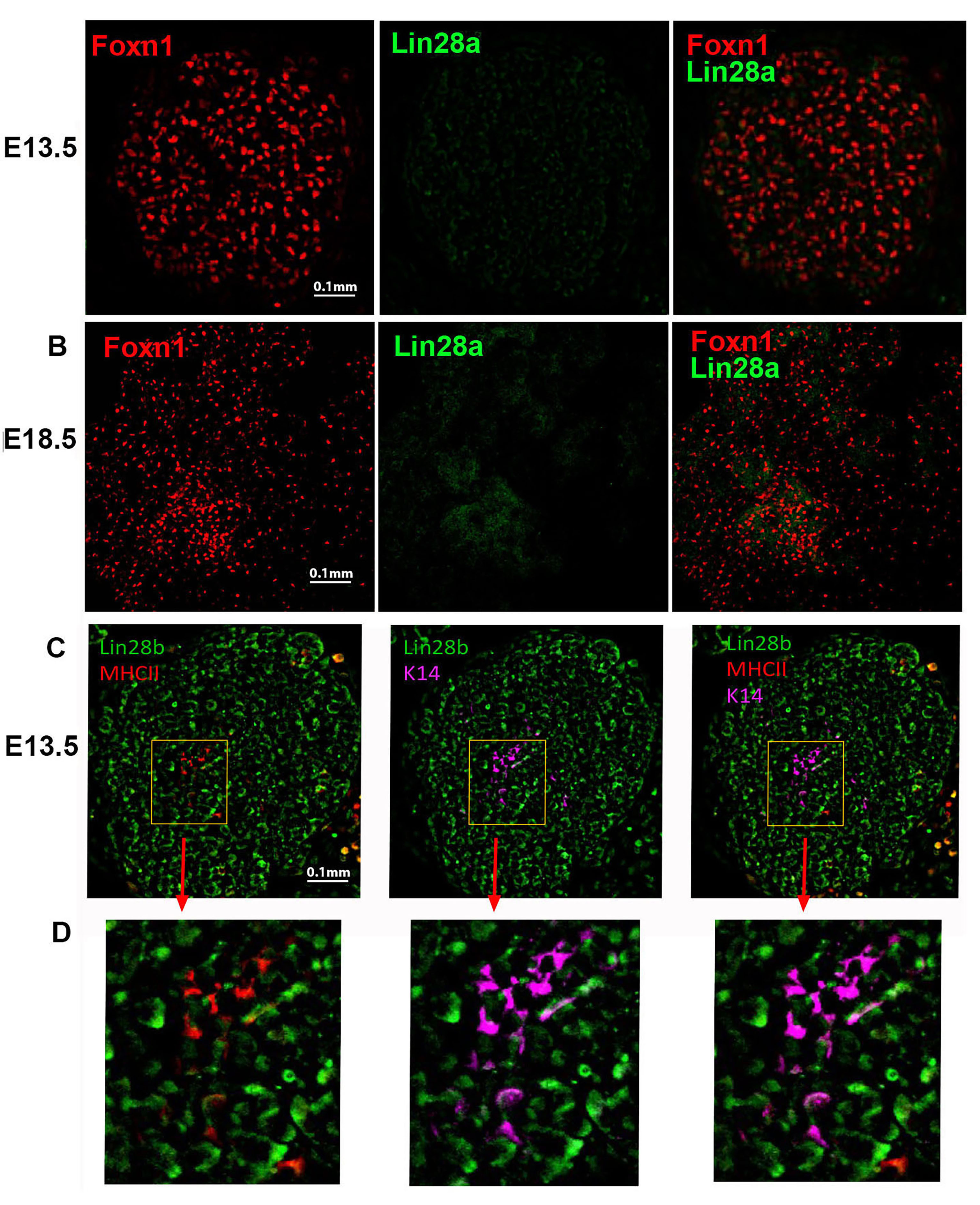

Supplement: Supplementary Figure 1 — Immunofluorescence staining of fetal thymic sections. (A, B). 4% PFA-fixed E13.5 (A) and E18.5 (B) thymic sections were stained by FOXN1 (red) and LIN28a (green, no positive signaling was detected on thymi). (C). Frozen E13.5 thymic sections were stained for LIN28b (green), MHCII (red), and K14 (pink). (D). The digitally enlarged images of the area indicated in C. Scale bar = 0.1 mm. [file Image_1.jpeg]

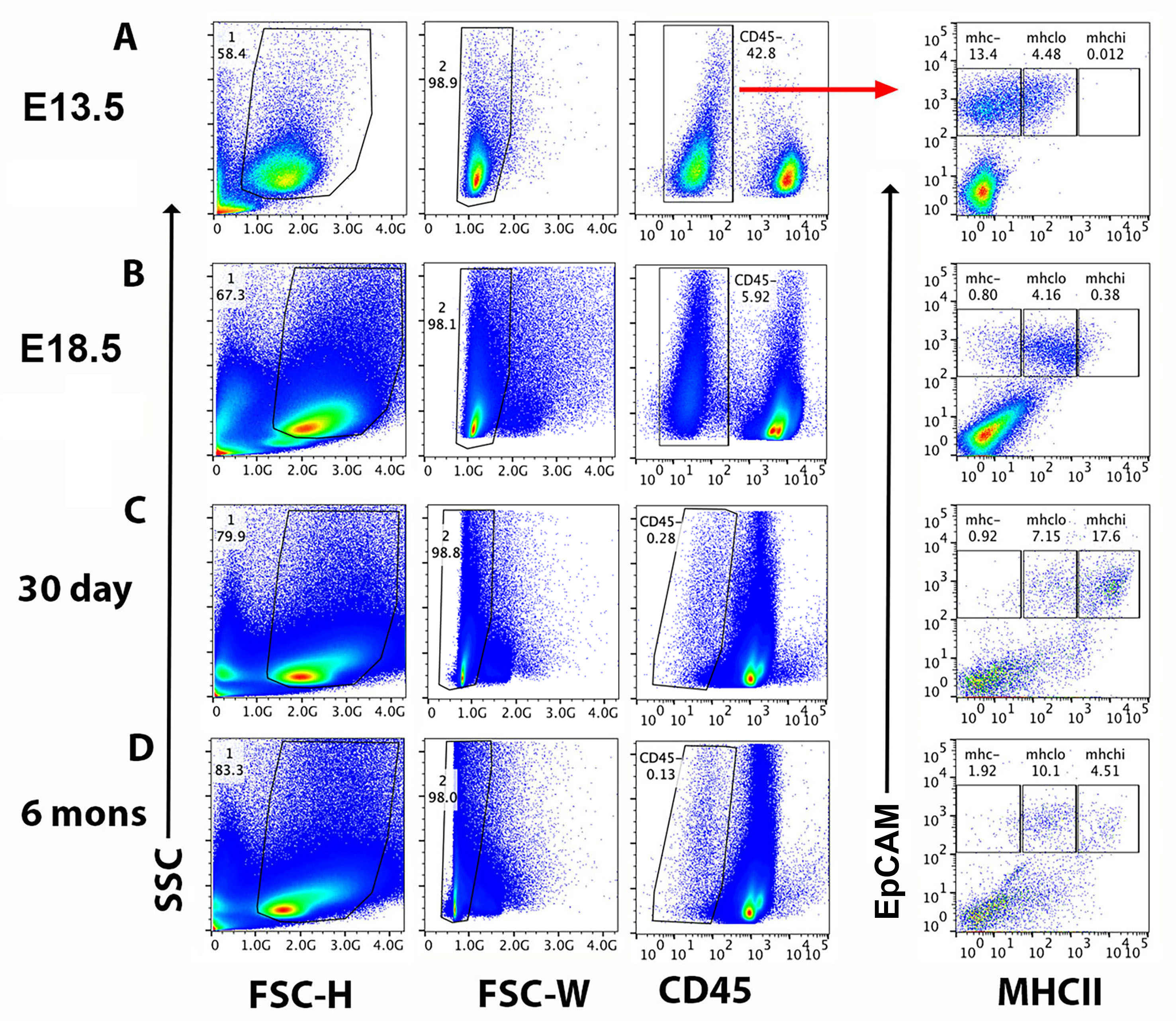

Supplement: Supplementary Figure 2 — TECs sorted from fetal and postnatal thymi. (A-D). The gate settings for TEC sorting (left). Representative profiles of EpCAM and MHCII staining and gates of EpCAM+ MHCII-, EpCAM+MHCIIlo, and/or EpCAM+MHCIIhi TCE subsets in the gated CD45- cells from thymi of fetal E13.5 (A), E18.5 (B), postnatal 30 days (C), and 6 months (D). [file Image_2.jpeg]

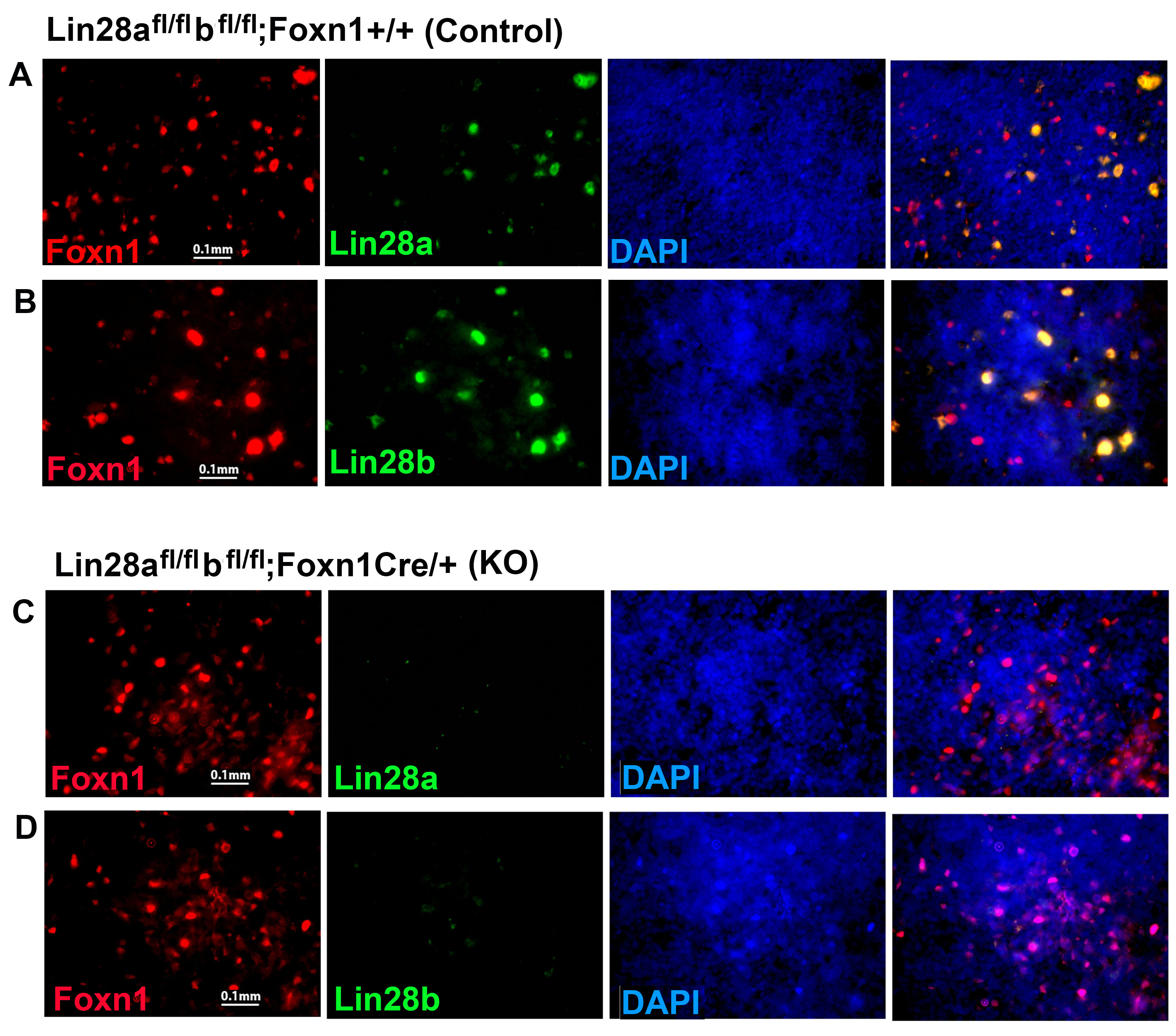

Supplement: Supplementary Figure 3 — Immunofluorescence staining of Lin28 a and b double KO thymic sections. (A, B). 4% PFA fixed Lin28afl/flbfl/fl;Foxn1+/+ control (2 month-old) thymic sections were stained by FOXN1 (red) and LIN28a (A) and LIN28b (B) (green). (C, D). 4% PFA fixed Lin28afl/flbfl/fl;Foxn1Cre/+ double KO (2 month-old) thymic sections were stained by FOXN1 (red) and LIN28a (C) and LIN28b (D) (green, no positive signaling was detected on thymi). Scale bar = 0.1mm. [file Image_3.jpeg]

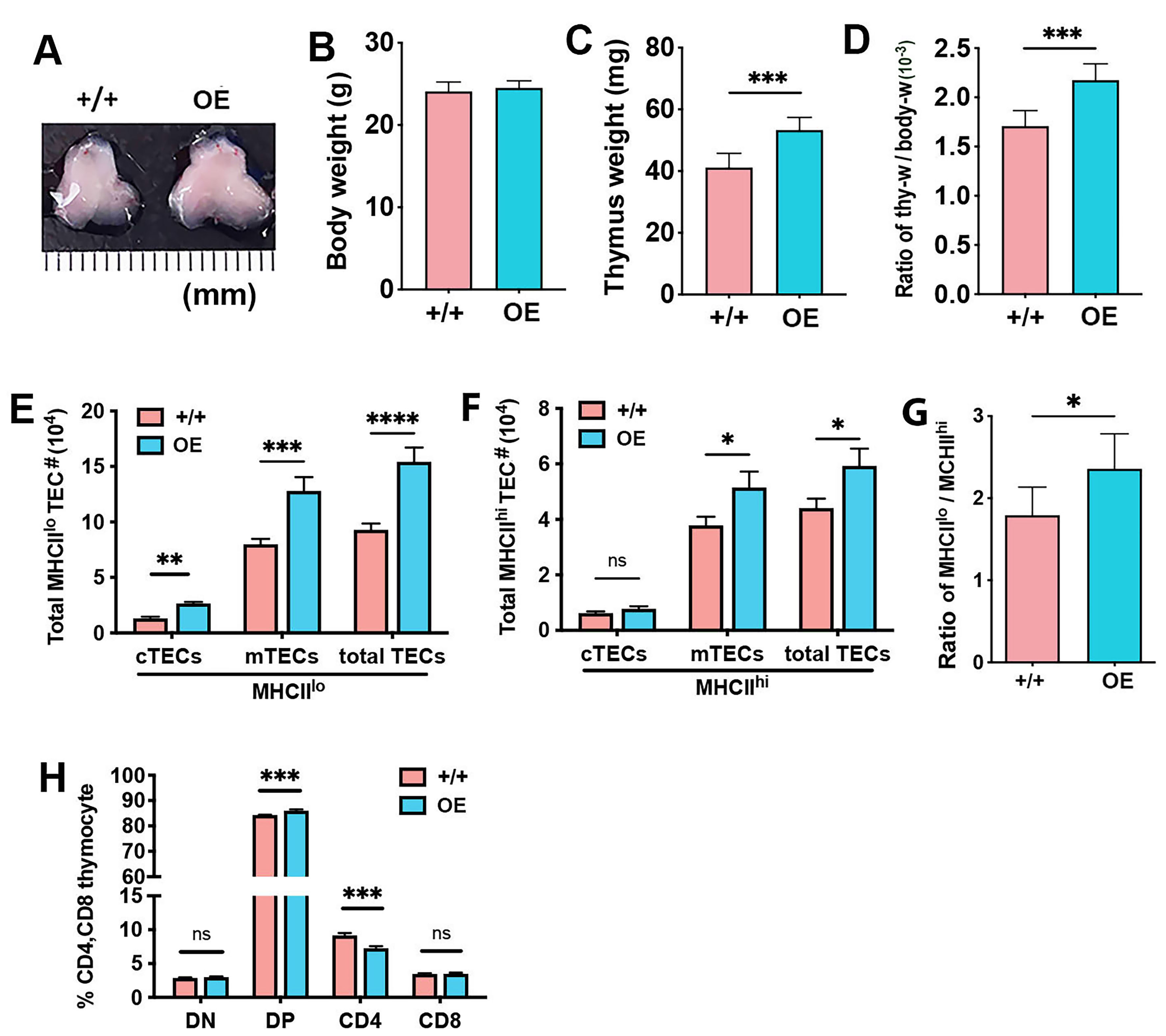

Supplement: Supplementary Figure 4 — TEC-specific overexpression of Lin28a caused an increase in thymus size in postnatal adult mice. (A). Image of thymi from 6-7-week-old iLin28a +/+ Wt (+/+) and iLin28a OE transgenic (OE) mice. (B). Mouse body weights of +/+ and OE mice. (C). Thymus weights of +/+ and OE mice. (D) Ratio of thymus weight vs body weight of +/+ and OE mice. (E). Total number of MHCIIlo TECs, including MHCIIlo cTECs and mTECs. (F). Total number of MHCIIhi TECs, including MHCIIhi cTECs and mTECs. (G). Ratio of MHCIIlo TECs vs MHCIIhi TECs. (H). Percentage of CD4 and CD8 thymocytes in total cells. Student’s t test (B-D) results between +/+ and OE mice: *P <0.05, **P <0.01, ***P <0.001, ****P <0.0001. ns: not significant. [file Image_4.jpeg]

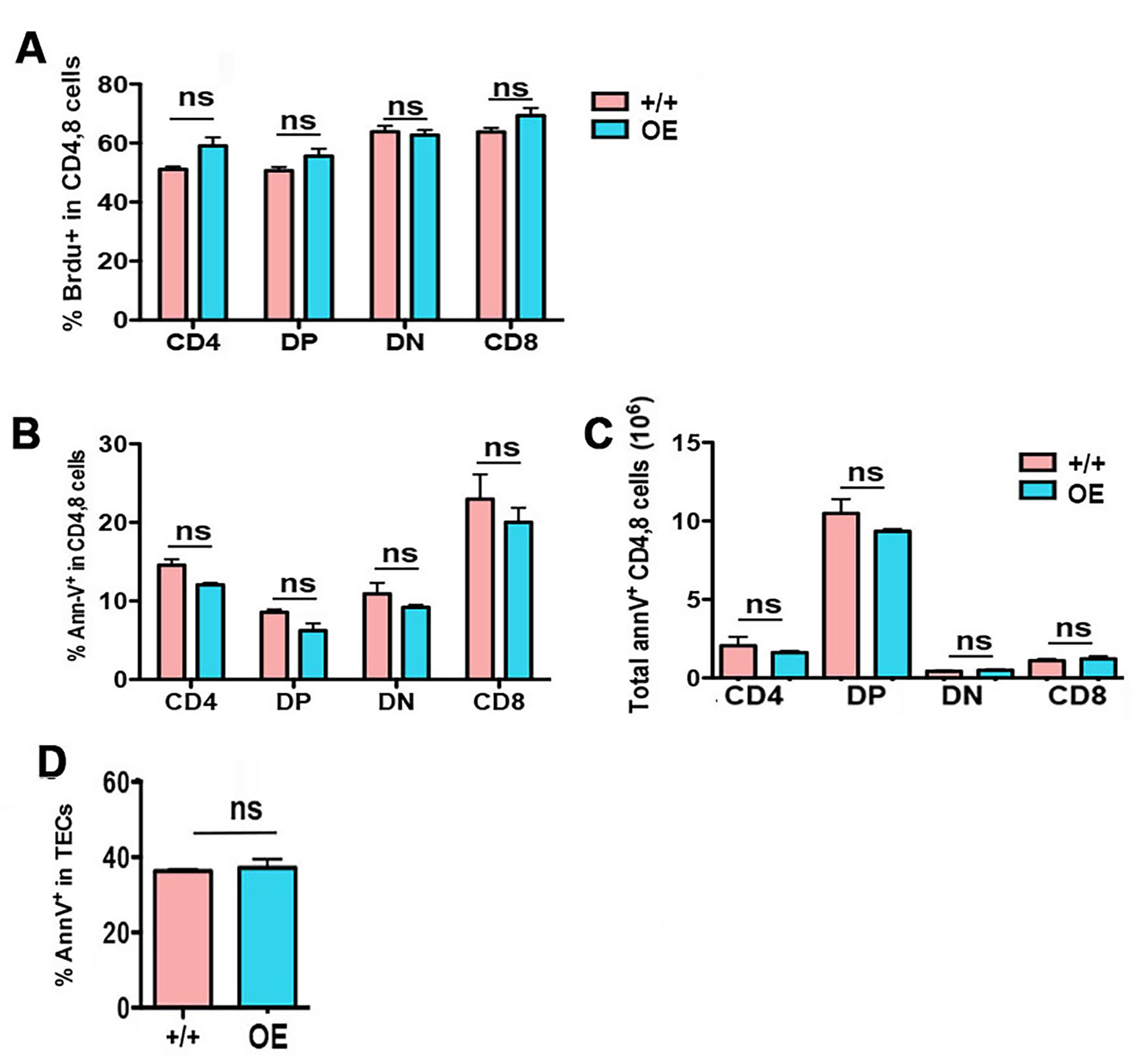

Supplement: Supplementary Figure 5 — BrdU and Annexin V staining of cells from TEC-specific overexpression of Lin28a in postnatal adult mice. (A). Percentage of BrdU+ cells in CD4 and CD8 thymocytes in +/+ and OE mice. (B). Percentage of Ann-V+ cells in CD4 and CD8 thymocyte in +/+ and OE mice. (C). Total Ann-V+ CD4 and CD8 thymocytes in +/+ and OE mice. (D). Percentage of Ann-V+ cells in total TECs in +/+ and OE mice. Data are representative of two individual experiments, (+/+, n=3, OE, n=4). Student’s t test (B-D) results between +/+ and OE mice:. ns: not significant. [file Image_5.jpeg]
